# Transglutaminase 2 Prevents Premature Senescence and Promotes Osteoblastic Differentiation of Mesenchymal Stem Cells through NRF2 Activation

**DOI:** 10.1155/2023/8815888

**Published:** 2023-10-20

**Authors:** Soo-Jin Lee, Ji-Woong Shin, Mee-Ae Kwon, Ki Baek Lee, Hyo-Jun Kim, Jin-Haeng Lee, Heun-Soo Kang, Jong Kwan Jun, Sung-Yup Cho, In-Gyu Kim

**Affiliations:** ^1^Department of Biochemistry and Molecular Biology, Seoul National University College of Medicine, Seoul, Republic of Korea; ^2^Department of Biomedical Sciences, Seoul National University College of Medicine, Seoul, Republic of Korea; ^3^Laboratory for Cellular Response to Oxidative Stress, Cell2in, Inc., Seoul, Republic of Korea; ^4^Department of Obstetrics and Gynecology, Seoul National University College of Medicine, Seoul, Republic of Korea; ^5^Cancer Research Institute, Seoul National University College of Medicine, Seoul, Republic of Korea; ^6^Medical Research Center, Genomic Medicine Institute, Seoul National University College of Medicine, Seoul, Republic of Korea; ^7^Institute of Human-Environment Interface Biology, Seoul National University College of Medicine, Seoul, Republic of Korea

## Abstract

Transglutaminase 2 (TG2) is a multifunctional enzyme that exhibits transamidase, GTPase, kinase, and protein disulfide isomerase (PDI) activities. Of these, transamidase-mediated modification of proteins regulates apoptosis, differentiation, inflammation, and fibrosis. TG2 is highly expressed in mesenchymal stem cells (MSCs) compared with differentiated cells, suggesting a role of TG2 specific for MSC characteristics. In this study, we report a new function of TG2 in the regulation of MSC redox homeostasis. During *in vitro* MSC expansion, TG2 is required for cell proliferation and self-renewal by preventing premature senescence but has no effect on the expression of surface antigens and oxidative stress-induced cell death. Moreover, induction of differentiation upregulates TG2 that promotes osteoblastic differentiation. Molecular analyses revealed that TG2 mediates tert-butylhydroquinone, but not sulforaphane, -induced nuclear factor erythroid 2-related factor 2 (NRF2) activation in a transamidase activity-independent manner. Differences in the mechanism of action between two NRF2 activators suggest that PDI activity of TG2 may be implicated in the stabilization of NRF2. The role of TG2 in the regulation of antioxidant response was further supported by transcriptomic analysis of MSC. These results indicate that TG2 is a critical enzyme in eliciting antioxidant response in MSC through NRF2 activation, providing a target for optimizing MSC manufacturing processes to prevent premature senescence.

## 1. Introduction

Mesenchymal stem cells (MSCs) are adult tissue stem cells that have a capacity for self-renewal and the potential to differentiate into osteoblasts, chondrocytes, adipocytes, and fibroblasts [1]. MSC has been isolated from many human tissues and were expanded *in vitro* to test their therapeutic efficacy in a wide range of animal models and clinical trials [2]. MSC therapy exhibited stimulation of tissue repair, suppression of inflammation, and modulation of immune responses by secreting a number of growth factors and cytokines rather than by providing differentiated cells [3]. However, *in vitro* MSC culture to obtain a clinical dose induces replicative senescence of MSCs and subsequent changes of stem cell characteristics, resulting in variable and inconsistent efficacy of MSC therapy among studies [4]. Thus, prevention or delay of MSC senescence during large-scale *in vitro* expansion is necessary for improving the efficacy of MSC therapy.

MSCs have been isolated from various tissues, including the umbilical cord, bone marrow, and adipocytes. Although MSCs were first discovered in bone marrow, bone marrow-derived MSCs present several limitations, such as their scarcity (0.001%–0.01%) [5, 6], reduced lifespan when isolated from aged donors [6, 7], and a highly painful collection procedure [6]. In the case of adipose-derived stem cells, the use of local anesthetic agents during isolation from adipose tissue has been reported to affect viability and yield [8]. In contrast, umbilical cord-derived MSCs (UC-MSCs) offer several advantages: (1) ethical and legal constraints are avoided during isolation from the human umbilical cord [9]; (2) tissue collection occurs after birth, ensuring the safety of both mother and fetus [10]; (3) UC-MSCs represent a more primitive progenitor population compared to other tissue sources [7]; (4) the placental barrier provides protection, reducing the risk of viral and bacterial infections [9]; (5) umbilical cord tissue harbors abundant MSCs that can be almost completely harvested [9]; and (6) UC-MSCs exhibit superior expansion capacity, enabling the generation of a sufficient number of cells for clinical transplantation within a short timeframe [9].

Cellular senescence is a state of irreversible cell-cycle arrest, and MSC becomes senescent after extensive replication due to erosion of telomeres [11]. In addition, when MSCs are cultured under normoxic conditions, increased production of reactive oxygen species (ROS) due to a metabolic shift from glycolysis to mitochondrial oxidative phosphorylation leads to premature senescence through activation of ATM-p53-p21 signaling pathway [12]. Besides, ROS also elicits the antioxidant response by activating the nuclear factor erythroid 2-related factor 2 (NRF2) signaling pathway [13]. Under homeostatic conditions, NRF2 binds to Kelch-like erythroid cell-derived protein with cap'n'collar homology-associated protein 1 (KEAP1)-Cul3 E3 ubiquitin ligase complex, leading to NRF2 ubiquitination and proteasomal degradation. An increase in ROS production induces oxidative modification of cysteine residues in KEAP1, causing the release of NRF2 from the complex and its translocation into the nucleus, wherein it upregulates the expression of antioxidant proteins, such as heme oxygenase 1 (*HMOX1*) and glutathione peroxidase, that contributes to protection of MSCs against oxidative damage [14]. Therefore, the NRF2 signaling pathway plays a critical role in the maintenance of redox homeostasis and protection of premature senescence during *in vitro* MSC expansion.

Transglutaminase 2 (TG2) is a calcium-dependent enzyme that modifies proteins by catalyzing transamidation reaction between glutamine residue and lysine residue of the substrates (protein crosslinking) or polyamines (polyamination), thereby modulating their functions [15, 16]. We have previously shown that oxidative stresses, exposure to hypoxia, UV-irradiation, and treatment with chemotherapeutics activate intracellular TG2, which inhibits I-kB and caspase 3 by forming their crosslinking products, resulting in eliciting inflammatory response, suppression of apoptosis, and remodeling of extracellular matrix [17–19]. Thus, oxidative stress-induced TG2 activation is implicated in the development of age-related diseases, including cataracts, pulmonary fibrosis, and UV-induced skin wrinkling [20–22]. In addition to the transamidation reaction, TG2 exhibits GTP hydrolyzing, kinase, and protein disulfide isomerase (PDI) activities [23]. It has been proposed that these TG2 activities may be involved in the regulation of signal transduction and mitochondrial function, although the interconversion of TG2 activities in the cells remains unknown.

TG2 is ubiquitously expressed in almost all cell types, including MSCs. Intriguingly, the level of TG2 expression in MSCs is 10–15 folds higher than that in differentiated osteoblasts, chondrocytes, adipocytes, and fibroblast cells [24], suggesting a role of TG2 in the regulation of MSC characteristics. In this study, we investigated the role of TG2 in MSCs by analyzing the effect of TG2 downregulation. Our results showed that TG2 increases cell proliferation and is also required for osteoblastic differentiation of human umbilical cord-derived MSCs. Molecular analyses revealed that TG2 mediates oxidative stress-induced NRF2 activation in a transamidase activity-independent manner. These results indicate that TG2 is a critical enzyme for eliciting antioxidant response, explaining increased senescent cells during *in vitro* expansion and defective osteogenic differentiation of TG2-deficient MSCs.

## 2. Materials and Methods

### 2.1. Isolation and Culture of Umbilical Cord-Derived MSCs

Human umbilical cords were obtained with informed consent from full-term births according to procedures approved by the Institutional Review Board of Seoul National University Hospital (IRB No. C-1708-083-878). Umbilical cords were removed immediately after cesarean section, washed with Dulbecco's phosphate-buffered saline (PBS), and umbilical cord vessels were removed. Single-cell suspension was prepared with an Umbilical Cord Dissociation Kit (Miltenyi Biotec, Germany) according to the manufacturer's instructions. Briefly, the umbilical cord was cut into small pieces and incubated in the enzyme solution provided in the kit at 37°C for 3 hr. Cells were separated with the gentleMACS Dissociator (Miltenyi Biotec, Germany). The remaining particles were removed with MACS SmartStrainer (100 *μ*m, Miltenyi Biotec, Germany). The cells were collected by centrifuge at 300 × *g* for 15 min at room temperature and suspended in *α*-MEM (Gibco, USA) supplemented with 10% fetal bovine serum (Gibco, USA) and 1% penicillin-streptomycin (Gibco, USA). MSCs were cultured in a humidified incubator with 5% CO_2_ at 37°C, and culture media were changed every 2 days. Cells collected after the third passage were used for the experiments. MSCs were characterized by flow cytometry. To induce hypoxia, MSCs were cultured in a BD GasPak EZ Anaerobe Gas Generating Pouch System (Cat. No. 260683; BD Biosciences, USA), which maintained the amount of oxygen to less than 1%.

### 2.2. Characterization of MSCs

The following primary antibodies were used for MSCs characterization: BV605-anti-human CD73 (Cat. No. 344024; Biolegend, USA), APC/Cy7-anti-human CD90 (Cat. No. 328132; Biolegend, USA), PE-anti-human CD105 (Cat. No. 323206; Biolegend, USA), BV711-anti-mouse/human CD44 (Cat. No. 103057; Biolegend, USA), BUV805-anti-human CD45 (Cat. No. 564914; Biolegend, USA), AlexaFluor 488-anti-human CD31 (Cat. No. 303110; Biolegend, USA), and BV-510-anti-mouse/human CD11b (Cat. No. 101263; Biolegend, USA). Flow cytometry was performed using LSR Fortessa with FACS DIVA software (BD Biosciences, USA), and stained cells were analyzed using Flowjo 7.6.

### 2.3. Bromodeoxyuridine (BrdU) Incorporation Assay

For the BrdU incorporation assay, cells were incubated with 10 *μ*M BrdU (Cat. No. 423401; Biolegend, USA) for indicated times. After fixing the cells with 70% ethanol, cells were permeabilized with 2 N HCl and 0.5% Triton X-100 for 30 min and then neutralized with 0.1 M sodium tetraborate for 10 min at RT. Cells were labeled with anti-BrdU antibody (Cat. No. 364108; Biolegend, USA) and analyzed by flow cytometry. Flow cytometry was performed using LSR Fortessa with FACS DIVA software (BD Biosciences, USA), and stained cells were analyzed using Flowjo 7.6.

### 2.4. Lentivirus Infection

Lentivirus expressing shRNA for TG2 was prepared by transfection of a mixture containing lentiviral and packaging vectors (ViraPower™ Lentiviral Packaging Mix, Thermo Fisher Scientific, USA) into HEK293FT cells using lipofectamine 3000. The virus was purified according to manufacturer's instructions. shRNA lentiviral construct for *TGM2* (TRCN0000000239, Sigma–Aldrich, USA) and pLenti CMV/TO Neo empty (w215-1, Addgene #17485) were used for generating lentiviral vectors. The shRNA lentiviral constructs were subcloned into a pLKO.1-puro backbone. The cDNAs of TG2 were cloned into pLEF-Puro lentiviral vector (a modification of pLL3.7, Addgene #11795) for lentivirus overexpressing TG2. Lentivirus particles were generated from HEK293FT cells with lentiviral packaging plasmid (psPAX2, Addgene #12260) and VSV-G envelope expressing plasmid (pMD2.G, Addgene #12259). MSCs were infected with lentivirus using 8 *μ*g/mL polybrene for 24 hr, and the knock-down and overexpression of TG2 were evaluated by western blotting. In our approach to generating a stable cell line, we omitted the intermediate puromycin selection and clone screening steps to avoid the necessity of multiple passages during the clonal selection process. We applied puromycin (1 *μ*g/mL) exclusively during the long-term culture of MSCs to assess cell proliferation.

### 2.5. Western Blot Analysis

Cell lysis, protein sampling, and western blot analysis were performed as described previously [25]. Cells were lysed with radioimmunoprecipitation assay buffer (50 mM Tris–Cl, pH 8.0, 150 mM NaCl, 0.1% sodium dodecyl sulfate (SDS), 1% Triton X-100, 0.5% sodium deoxycholate) with a protease inhibitor cocktail (Roche, Swiss). Total proteins were quantitated by BCA assay. Equal amounts of protein were separated by SDS–PAGE (polyacrylamide gel electrophoresis) and transferred onto a nitrocellulose membrane. The membranes were blocked with 5% skim milk dissolved in TBS-T (Tris-buffered saline, 0.1% Tween 20) for 1 hr, then incubated with primary antibodies at 4°C overnight. After incubating with an horseradish peroxidase (HRP)-conjugated secondary antibody, immunoreactive proteins were detected with an enhanced chemiluminescence (ECL) substrate (Thermo Fisher Scientific, USA). Following antibodies were used: anti-NRF2 (Cat. No. ab62352; Abcam, UK), anti-HMOX1 (Cat. No. sc-136960; Santa Cruz, USA), anti-NQO1 (Cat. No. sc-32793; Santa Cruz, USA), anti-GAPDH (Cat. No. sc-47724; Santa Cruz, USA), anti-Histone H3 (Cat. No. 9715; Cell Signaling Technology, USA), anti-HIF-1*α* (Cat. No. 36169; Cell Signaling Technology, USA), anti-p16 (Cat. No. 92803; Cell Signaling Technology, USA) and anti-*β*-actin (Cat. No. A5441, Sigma–Aldrich, USA). The monoclonal antibody against TG2 was prepared as described previously [26].

### 2.6. Measurement of MSC Proliferation

The proliferation rate of MSC was assessed by calculating the cumulative population doubling level (CPDL) as the following equation. CPDL = ln(Nf/Ni)/ln2, where Nf and Ni are the number of cells at the end of each passage and at initial seeding, respectively. In total, 50,000 cells were plated in quadruplicate in a 6-well culture plate (Corning, USA) and maintained for 3 days. The cell numbers were counted using a hemocytometer and 0.4% trypan blue dye at the end of each passage. During the successive passages, the cell culture media was supplemented with a concentration of 1 *μ*g/mL puromycin to enrich the lentivirus-infected cells.

### 2.7. Colony Forming Unit—Fibroblast Assay

MSCs were plated at 2,500 cells per well in 6-well culture plates (Corning, USA) and cultured for 9 days at 37°C in a 5% humidified CO_2_ incubator, with media changes performed every 3 days. Dishes were washed with PBS and fixed with 100% methanol for 5 min. Cells were stained with 0.1% crystal violet in methanol with gentle shaking for 20 min at room temperature.

### 2.8. SA-*β*-GAL Staining

Cells were fixed with 4% paraformaldehyde for 5 min at room temperature after being washed with PBS twice. Subsequently, the cells were washed again with PBS and incubated with SA-*β*-gal staining solution (40 mM citric acid, pH 6, 5 mM potassium ferrocyanide, 5 mM potassium ferricyanide, 150 mM NaCl, 2 mM MgCl_2_) and 1 mg/mL X-gal at 37°C for 16 hr to visualize the staining.

### 2.9. In Vitro Differentiation of MSCs

MSCs were cultured until 70% confluency in 6-well culture plates (Corning, USA). Differentiation was induced by replacing the culture medium with either osteogenic (Cat. No. A1007201; Gibco, USA) or adipogenic (Cat. No. A1007001; Gibco, USA) differentiation medium. The cells were cultured for 3 weeks at 37°C in a humidified 5% CO_2_ incubator, with the differentiation medium changed every 3 days. Afterward, the cells were harvested for RNA purification. Osteoblasts were stained with Alizarin red S staining solutions to identified calcium depositions. Alizarin Red S staining was performed following the manufacturer's instructions (Cat. No.8678; ScienCell, USA). Briefly, cells were washed with PBS and fixed with 4% formaldehyde for 15 min at room temperature. After being washed three times with distilled water, cells were stained with 40 mM of Alizarin red S staining solution and gently shaken for 30 min. Subsequently, cells were washed five times with distilled water. Adipocytes were stained with oil red O to visualize intracellular lipid droplets. Oil red O powder (Cat. No. O0625; Sigma–Aldrich, USA) was dissolved in 100% isopropanol to create a 0.3% oil red O solution. Cells were washed twice with PBS and fixed with 4% formaldehyde for 30 min at room temperature. Next, the cells were stained with 0.3% oil red O solution in 60% isopropanol, in darkness, for 30 min at room temperature. After removing the oil red O dye, cells were washed more than two times with distilled water and visualized using a light microscope.

### 2.10. Real-Time Quantitative PCR (RT-qPCR)

Total RNA was purified with Total RNA Kit I (Cat. No. R6834-02; Omega Bio-Teck, USA). For the reverse transcription reaction, 1 *μ*g of total RNA was utilized with oligo dT and reverse transcriptase (Invitrogen, USA). qPCR was performed with CFX96 Real-Time System (Bio-Rad, USA) and 2 × qPCR Master Mix (Cat. No. KK4602; Kapa Biosystems, USA). The relative mRNA expression was calculated by the 2^−*ΔΔ*Ct^ method [27]. Sequences of primers are listed in Table S1.

### 2.11. *In Situ* TG Activity Assay

*In situ* TG activity was assayed by estimating the amount of 5-biotinamidopentylamine (BP, Cat. No. 21345; Thermo Fisher Scientific, USA) incorporated into the cellular proteins. MSCs were incubated with 1 mM BP for 1 hr and harvested by centrifugation. The cell lysates were prepared using lysis buffer (containing 50 mM Tris–HCl pH 7.4, 1% NP40, 0.25% sodium deoxycholate, 1 mM ethylenediamine tetraacetic acid (EDTA)), followed by centrifugation (14,000 *× g*, 10 min at 4°C). After quantitating the protein concentration, protein samples were boiled in sample loading buffer (containing 60 mM Tris–HCl pH 6.8, 25% glycerol, 2% SDS, 14.4 mM 2-mercaptoethanol, 0.1% bromophenol blue) for 10 min, separated on SDS–PAGE, and transferred onto nitrocellulose membranes. The BP-incorporated proteins were probed using streptavidin coupled to HRP (Cat. No. 21126; Thermo Fisher Scientific, USA) and were detected with ECL substrate (Thermo Fisher Scientific, USA).

### 2.12. Luciferase Reporter Assay

HEK293FT cells were plated at 4 × 10^4^ cells in a 48-well plate. The cells were transfected with a vector expressing constitutively active NRF2 (CA-NRF2; 100 ng), 8 × antioxidant responsive element (ARE)-driven luciferase vector (150 ng) and pRL-TK vector (50 ng) using lipofectamine 3000 (Invitrogen, USA). The pRL-TK vector was cotransfected to normalize luciferase activity. Following transfection, the cells were treated with tert-butylhydroquinone (tBHQ; 25 *μ*M) for 48 hr. Luciferase reporter activity was measured by Dual Luciferase Assay Kit (Promega, USA) in accordance with the manufacturer's instructions.

### 2.13. Nucleus Fractionation

Nucleus fractionations were performed according to the Nuclear Fractionation Protocol (Abcam, UK). Briefly, MSCs were harvested at indicated times after treatment with tBHQ (25 *μ*M). The cells were mixed with 400 *μ*L of lysis buffer (10 mM HEPES, 1.5 mM MgCl_2_, 10 mM KCl, 0.5 mM DTT, 0.05% NP40, pH 7.9, containing protease and phosphatase inhibitor cocktail), thoroughly scraped and incubated on ice for 10 min. The mixture was centrifuged for 10 min at 3,000 rpm, 4°C. The supernatant contains the cytoplasmic fraction. Next, the nuclear pellets were lysed with 120 *μ*L of buffer (5 mM HEPES, 1.5 mM MgCl_2_, 0.2 mM EDTA, 0.5 mM DTT, 26% glycerol, pH 7.9, containing protease and phosphatase inhibitor cocktail). 4 M NaCl was added to make its concentration 300 mM and the homogenized pellet was incubated on ice for 30 min at 14,000 *× g*, 4°C, and the resulting supernatant contains the nuclear fraction. Each fraction was quantified with western blotting.

### 2.14. Cell Viability Assay

Cells were prepared by seeding 8,000 cells in a 96-well plate and treated with hydrogen peroxide, tert-butyl hydroperoxide (tBHP), and RAS-selective lethal 3 (RSL3) in serum-free media for 24 hr. Plates were incubated for 24 hr at 37°C in a humidified 5% CO_2_ incubator. To estimate cell viability, 10 *μ*L of CCK-8 solutions (Cell Counting Kit-8, Dojindo, Japan) was added to each well of the plate. After incubated the plate for 3 hr, the absorbance was measured at 450 nm.

### 2.15. Transcriptome Analyses

Sequencing data of human adipose-derived MSCs after osteogenic differentiation (GSE159138) were downloaded from GEO (Gene Expression Omnibus; https://www.ncbi.nlm.nih.gov/geo/). Gene expression levels were quantified as fragments per kilobase of exon per million mapped reads. Microarray data of MSCs from the bone marrow of 61 different donors (GSE 39540) were also downloaded from GEO. Each Affymetrix dataset was background-adjusted and normalized by the Robust Multichip Averaging algorithm in the Affy package using R ver. 3.6.0 [28]. The genes whose expressions were positively correlated with TG2 expression were determined by Pearson correlation. The ClueGO plug-in (v2.5.2, http://www.ici.upmc.fr/cluego/) in the Cytoscape software (v3.6.1, http://cytoscape/org/) was used to analyze gene ontology (GO) and functional groups in networks for positively correlated genes with TG2 [29]. The GO Biological Process database (http://www.geneontology.org/) was used for functional enrichment analysis. Significantly enriched GO terms were calculated using a two-sided hypergeometric test with a Bonferroni correction (*P* < 0.05), and the degree of connectivity between terms in the network was calculated using kappa statistics (kappa score of 0.4).

### 2.16. Statistical Analysis

GraphPad Prism 5.0 statistical software (GraphPad Software, USA) was used for statistical evaluations. Differences between the two variables were assessed by Student's *t*-test. *P* < 0.05 was considered statistically significant. Error bars represent mean ± SEM.

## 3. Results

### 3.1. TG2 Is Required for Cell Proliferation but Has No Effect on Surface Marker Expression in Human Umbilical Cord-Derived MSCs

**Figure 1:**
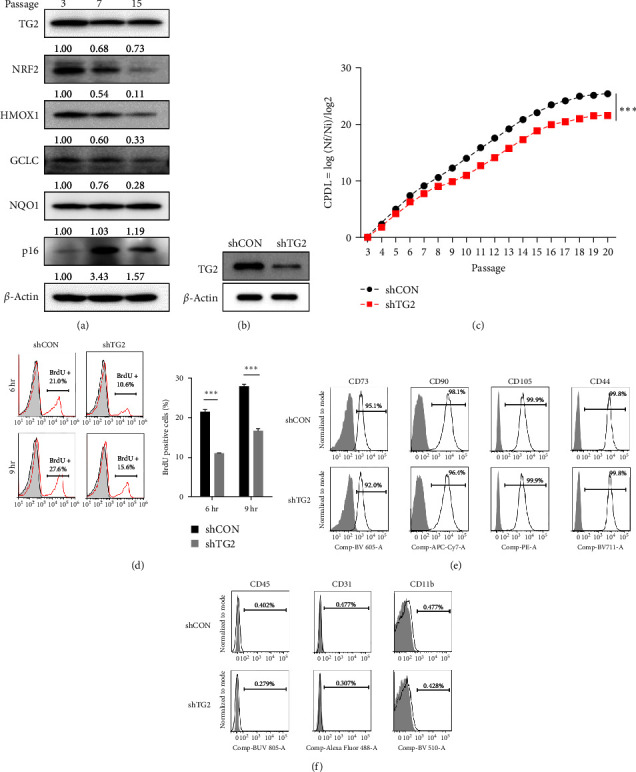
TG2 is required for cell proliferation but has no effect on surface marker expression in human umbilical cord-derived MSCs. (a) Human umbilical cord-derived MSCs were induced into replicative senescence after subculture for a long-term expansion (passage number 3, 7, and 15). The expression levels of TG2, NRF2, NRF2-responsive genes, and senescence marker (p16) were assessed by western blot analysis. *β*-Actin was used as the loading control, and the relative intensities were normalized using the loading control. (b) MSCs were transfected with lentivirus expressing shRNA for TG2. The level of TG2 protein was assessed by western blot analysis. *β*-Actin was used as the loading control in the experiment. (c, d) Cell proliferation rate of wild-type and TG2-knockdowned MSCs was estimated by measuring cumulative population doubling level (CPDL) through passage 3–20 (c) and the percentage of BrdU incorporated cells with flow cytometry (d). (e, f) Representative examples of flow cytometric profiles from wild-type and TG2-knockdowned MSCs. The percentage of cells expressing positive surface markers ((e), CD73, CD90, CD105 and CD44) and negative markers ((f), CD45, CD31, and CD11b) for MSCs was shown. The results for western blots are represented from three independent experiments. The measurement of growth rate in MSCs and flow cytometry images are representative results from three independent experiments performed. Statistical significance was tested by Student's *t*-test (*n* = 3;  ^*∗∗∗*^*P* < 0.001).

To investigate the role of TG2 in MSCs, we conducted a long-term culture of MSCs isolated from the human umbilical cord. Notably, we observed a decrease in TG2 protein levels corresponding to passage numbers (Figure 1(a)), which was consistent with the decline in the expression of stemness-related genes, including NRF2 and HMOX1, and the increase in the expression of the senescence marker, p16. Next, we assessed whether TG2 downregulation affects the biological characteristics of MSCs. To this end, MSCs isolated and cultured from the human umbilical cord (passage number 3) were transfected with lentivirus expressing shRNA for TG2. Western blot analysis showed the decreased level of TG2 protein in shTG2-transfected MSCs compared with control cells (Figure 1(b)). We noted that TG2-downregulated MSCs required more time to reach confluence for cell splitting than control cells, suggesting that TG2-depletion reduced MSC proliferation. To confirm this observation, we estimated the MSC proliferation rate and found that the calculated CPDL of TG2-downregulated MSCs was significantly reduced compared to those of control MSCs through passages 3–20 (Figure 1(c)). Moreover, flow cytometry analysis showed that the percentage of BrdU-incorporated cells in TG2-downregulated MSCs was significantly lower than those in control cells (Figure 1(d)). We then examined whether TG2 downregulation alters the expression of surface markers for MSCs proposed by the Mesenchymal and Tissue Stem Cell Committee of the International Society for Cellular Therapy [30]. Flow cytometry analysis showed that there was no difference between TG2-downregulated and control MSCs in the expression of CD73, CD90, CD105, and CD44, which are positive markers for MSCs (Figure 1(e)), and CD45, CD31, and CD11b, which are negative markers for MSCs (Figure 1(f)). These results indicate that TG2 is required for promoting the proliferation of MSCs but dispensable in surface marker expression of MSCs.

### 3.2. MSCs with Downregulated TG2 Are Prone to Premature Senescent Induction, but Not Oxidative Stress-Induced Apoptosis during In Vitro Cell Expansion

**Figure 2:**
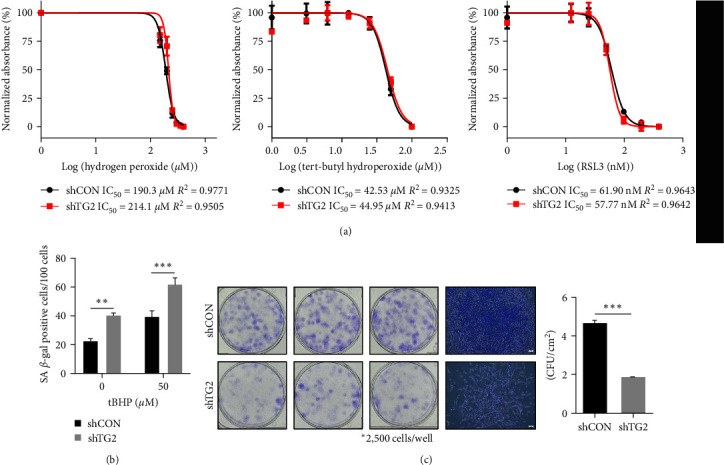
MSCs with downregulated TG2 are prone to premature senescent induction but not oxidative stress-induced apoptosis during *in vitro* cell expansion. (a) Wild-type and TG2-knockdowned MSCs were treated with H_2_O_2_, tert-butylhydroperoxide (tBHP), and RAS-selective lethal 3 (RSL3) for 24 hr at indicated concentrations. Cell viability was evaluated by WST assay. (b) Wild-type and TG2-knockdowned MSCs were treated with tBHP (50 *μ*M) for 24 hr and stained for SA-*β*-galactosidase. (c) The self-renewal capacity of wild-type and TG2-knockdowned MSCs was assessed by colony-forming unit (CFU) assay. Crystal violet-staining was performed on day 9. The pattern of colonies was observed with microscopy. The scale bar indicates 100 *µ*m. Data is shown as the mean values ± SEM from three independent experiments, each with technical replicates. Statistical significance was tested by Student's *t* test (*n* = 3;  ^*∗∗*^*P* < 0.01;  ^*∗∗∗*^*P* < 0.001).

MSCs cultured *in vitro* exhibit increased ROS generation due to enhanced mitochondrial oxidative metabolism [31], inducing apoptotic cell death or premature senescence of MSCs. Previous reports showed that TG2 suppresses oxidative stress-induced apoptosis of tumor cells through caspase-3 inhibition [18]. To test whether the antiapoptotic function of TG2 contributes to MSC proliferation, we compared apoptotic sensitivity to oxidative stresses between control and TG2-downregulated MSCs. Cells were treated with hydrogen peroxide, tBHP, and RSL3, an inhibitor of glutathione peroxidase. The MTT assay revealed that TG2 downregulation had no effect on IC_50_ values for cell death by oxidative stress-inducing agents (Figure 2(a)), suggesting that unlike in tumor cells, TG2 has no role in protection from oxidative stress-induced cell death in MSCs. To further evaluate the effect of oxidative stress on TG2-downregulated MSCs, we examined senescence induction of MSCs by senescence-associated *β*-galactosidase (SA-*β*-gal) staining and found that TG2 downregulation significantly increases SA-*β*-gal-positive cells even under normal culture conditions as well as after tBHP treatment (Figure 2(b)). Because senescent cells lost their ability to proliferate, we assessed the self-renewal capacity of MSC. Consistently, the number of colony-forming unit fibroblasts and colony cell density of TG2-downregulated MSCs was significantly reduced compared with that of control MSCs (Figure 2(c)). These results indicate that TG2 protects MSCs from the induction of premature senescence during *in vitro* expansion.

**Figure 3:**
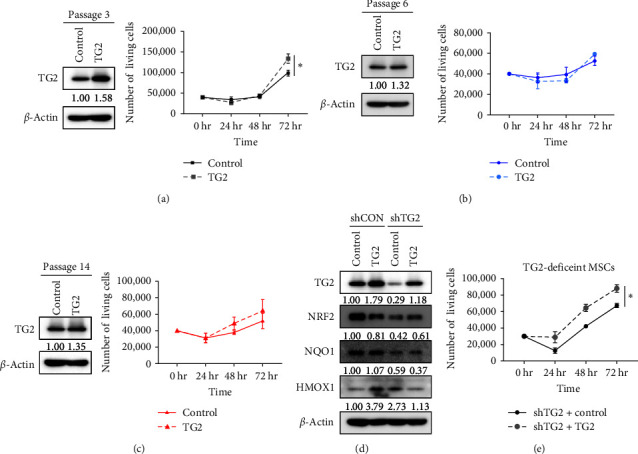
TG2 promotes MSC proliferation during early passages. (a–c) MSCs were transduced with TG2 overexpression or a control vector at passage 3 (a), passage 6 (b), and passage 14 (c). Cell viability assays were performed using trypan blue staining. (d) The expression levels of TG2, NRF2, NQO1, and HMOX1 upon the reintroduction of TG2 into TG2-deficient MSCs. The protein expression levels were analyzed using western blotting. The loading control used was *β*-actin. The western blots are representative results from three independent experiments performed. (e) Cell proliferation upon the reintroduction of TG2 into TG2-deficient MSCs. Cell viability was assessed through the trypan blue staining. The data are shown as the average values along with the standard error of the mean (SEM) obtained by conducting three technical replicates within a single experiment. Statistical significance was tested by Student's *t*-test (*n* = 3;  ^*∗*^*P* < 0.05).

We also performed an overexpression experiment of TG2 in MSCs using lentivirus transduction. We found that overexpression of TG2 increased the proliferation of MSCs in the early passage MSCs (passage 3) but not in late passage MSCs (passage 6, 14) (Figure 3(a)–3(c)). In addition, upon re-introducing TG2 in TG2-deficient MSCs at passage 3, we observed an increase in the protein expression of NRF2, a stemness-related gene (Figure 3(d)), and a significant enhancement in the proliferation of TG2-deficient MSCs (Figure 3(e)). These results suggest that TG2 enhances MSC proliferation during early passages, but its proliferation-promoting activity diminishes in later passages. This could be attributed to the characteristics of senescence, which is an irreversible process that arises due to telomere erosion after a specific number of cell divisions [32].

### 3.3. TG2 Is Required for Osteoblastic, but Not Adipocytic Differentiation of MSCs

**Figure 4:**
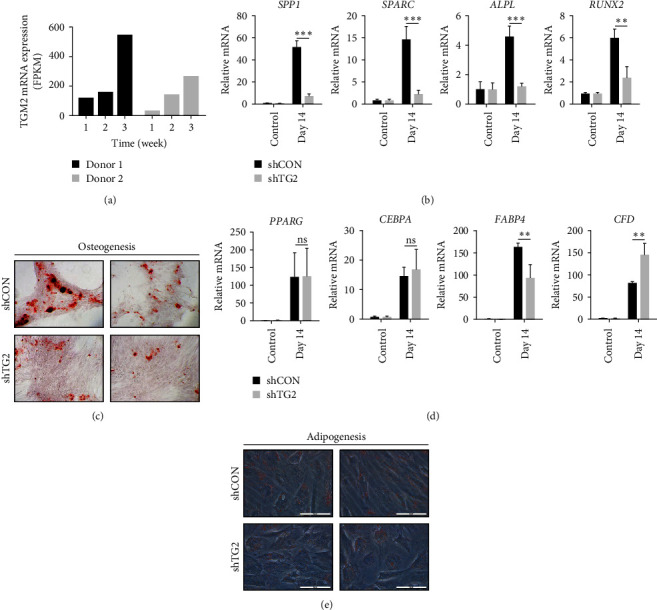
TG2 is required for osteoblastic but not adipocytic differentiation of MSC. (a) The mRNA level of TG2 after induction of osteoblast differentiation was assessed using RNA sequencing data from the Gene Expression Omnibus (GEO) database (GSE159138). (b) The mRNA levels of osteoblast markers (*SPP1*, *SPARC*, *ALPL*, and *RUNX2*) in wild-type and TG2-knockdowned MSCs. The mRNA levels were measured by real-time PCR after 2-week culture in differentiation-inducing media. (c) Staining for osteogenic differentiation. After a 3-week culture in differentiation-inducing media, Alizarin red staining was used to characterize osteoblasts that were differentiated from control or TG2-knockdowned MSCs. (d) The mRNA levels of adipocyte markers (*PPARG*, *CEBPA*, *FABP4*, and *CFD*) in wild-type and TG2-knockdowned MSCs. The mRNA levels were measured by real-time PCR after 2-week culture in differentiation-inducing media. (e) Staining for adipogenic differentiation. After a 3-week culture in differentiation-inducing media, oil red O staining was used to characterize adipocytes that were differentiated from control or TG2-knockdowned MSCs. The real-time PCR assay was repeated in three independent experiments, and representative results were presented. The information is presented as the mean values and their corresponding SEM. These values were derived from three technical replicates performed within a single experiment. Statistical significance was tested by Student's *t*-test (*n* = 3;  ^*∗∗*^*P* < 0.01;  ^*∗∗∗*^*P* < 0.001).

TG2-knockout mice exhibit lower bone mass due to increased osteoclast formation [33, 34]. To investigate the role of TG2 in differentiation into osteoblasts, we first evaluated the expression level of TG2 during osteoblast differentiation of MSC using RNA sequencing data from the GEO database (GSE159138) and found that the levels of TG2 mRNA were rapidly increased after induction of osteoblast differentiation (Figure 4(a)). We then evaluated the effect of TG2-downregulation on the expression levels of differentiation markers in MSCs after 2 weeks of culture in differentiation-inducing media. When osteoblastic differentiation was induced, the expression levels of osteoblast markers, secreted phosphoprotein 1 (*SPP1*), secreted protein acidic and cysteine-rich (*SPARC*), alkaline phosphatase (*ALPL*), and RUNX family transcription factor 2 (*RUNX2*) were significantly reduced in TG2-downregulated MSCs compared with those of control cells (Figure 4(b)). In addition, following a 3-week incubation in differentiation-inducing media, Alizarin red staining revealed a reduction in osteogenic differentiation in TG2-downregulated MSCs (Figure 4(c)). During induction of adipocytic differentiation, by contrast, TG2-downregulation had no effect on the level of peroxisome proliferator-activated receptor gamma (*PPARG*) and CCAAT enhancer binding protein alpha (*CEBPA*), decreased fatty acid binding protein 4 (*FABP4*) expression or increased complement factor D (*CFD*) expression (Figure 4(d)), which was consistent with adipogenic differentiation estimated by oil red O staining (Figure 4(e)). Thus, TG2 upregulation is required for osteoblastic differentiation of MSCs but not for adipocytic differentiation, implying that TG2 regulates MSC differentiation in a lineage-dependent manner.

### 3.4. TG2 Mediates Tert-Butylhydroquinone, but Not Sulforaphane, -Induced NRF2 Activation

**Figure 5:**
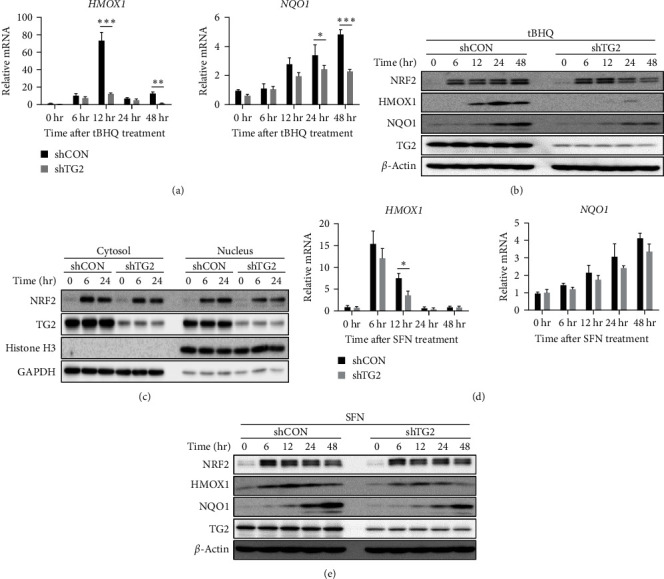
TG2 mediates tert-butylhydroquinone, but not sulforaphane, -induced Nrf2 activation. (a, b) Wild-type and TG2-knockdowned MSCs were treated with tert-butylhydroquinone (tBHQ, 25 *μ*M) for the indicated period of time. The expression levels of heme oxygenase 1 (*HMOX1*) and quinone oxidoreductase 1 (*NQO1*) were estimated by real-time PCR (a) and western blot analysis (b). *β*-Actin served as the loading control in the experiment. (c) Lysates prepared from wild-type and TG2-knockdowned MSCs treated with tBHQ (25 *μ*M) were separated into cytosolic and nuclear fractions. Protein levels of NRF2 in each fraction were estimated by western blot analysis. GAPDH and histone H3 were used as cytoplasmic and nuclear marker proteins, respectively. (d, e) Wild-type and TG2-knockdowned MSCs were treated with sulforaphane (SFN, 10 *μ*M) for the indicated period of time. The expression levels of heme oxygenase 1 (*HMOX1*) and quinone oxidoreductase 1 (*NQO1*) were estimated by real-time PCR (d) and western blot analysis (e). The western blots and real-time PCR images are representative results from three independent experiments performed. Statistical significance was tested by Student's *t*-test ( ^*∗*^*P* < 0.05;  ^*∗∗*^*P* < 0.01;  ^*∗∗∗*^*P* < 0.001).

Premature senescence can be prevented by activating NRF2, a master regulator of redox homeostasis [35]. Moreover, NRF2 plays a critical role in the proliferation, stemness, and osteogenesis of human umbilical cord-derived MSCs by upregulating the transcription of various genes [36, 37]. To account for the role of TG2 in MSC senescence and differentiation, we sought to examine the effect of TG2 downregulation on oxidative stress-induced NRF2 activation. MSCs were treated with tBHQ, an activator of NRF2 by inducing mitochondrial oxidative stress [38], and the expression levels of NRF2-responsive genes were estimated by RT-PCR and western blot analysis. Treatment with tBHQ induced a gradual increase of mRNA as well as protein levels of HMOX1 and quinone oxidoreductase 1 (NQO1) in control MSCs, but less in TG2-downregulated MSCs (Figures 5(a) and 5(b)). Similarly, tBHQ treatment caused an increase of NRF2 protein in the whole lysate (Figure 5(b)) as well as in the nucleus (Figures 5(c)) of control MSCs, but less of TG2-downregulated MSCs (Figures 5(b) and 5(c)). These results indicate that TG2 augments the antioxidant response of MSCs by stabilizing NRF2.

ROS induces the oxidation of KEAP1 cysteine residues that disrupt NRF2-KEAP1 interaction, thereby protecting NRF2 from proteasomal degradation [39]. However, our results showed that tBHQ treatment failed to increase NRF2 protein level in the absence of TG2 (Figure 5(b)), suggesting that TG2 may be required for the disruption of NRF2-KEAP1 interaction following oxidation of KEAP1 cysteine residues. To test whether oxidized KEAP1 at cysteine residues is a target for TG2 activity, we assessed the effect of protecting cysteine residues from ROS-induced oxidation on TG2-mediated NRF2 stabilization. MSCs were treated with sulforaphane (SFN), an isothiocyanate that reversibly reacts with KEAP1 cysteine residues, producing dithiocarbamate adducts [40], and NRF2 protein level and activity were monitored. The levels of *HMOX1* and *NQO1* mRNA and protein were increased in both control and TG2-downregulated MSCs, and there was no statistically significant difference between both cell types (Figures 5(d) and 5(e)). When compared with tBHQ-treated MSCs, TG2 downregulation did not diminish the protein level of NRF2 in SFN-treated cells (Figure 5(e)). These results indicate that TG2 stabilizes NRF2 by modifying oxidized cysteine residues of KEAP1.

### 3.5. TG2 Activates NRF2 in a Transamidase Activity-Independent Manner and the Upregulation of TG2 under Hypoxic Conditions Is Required for the Proliferation of MSCs

**Figure 6:**
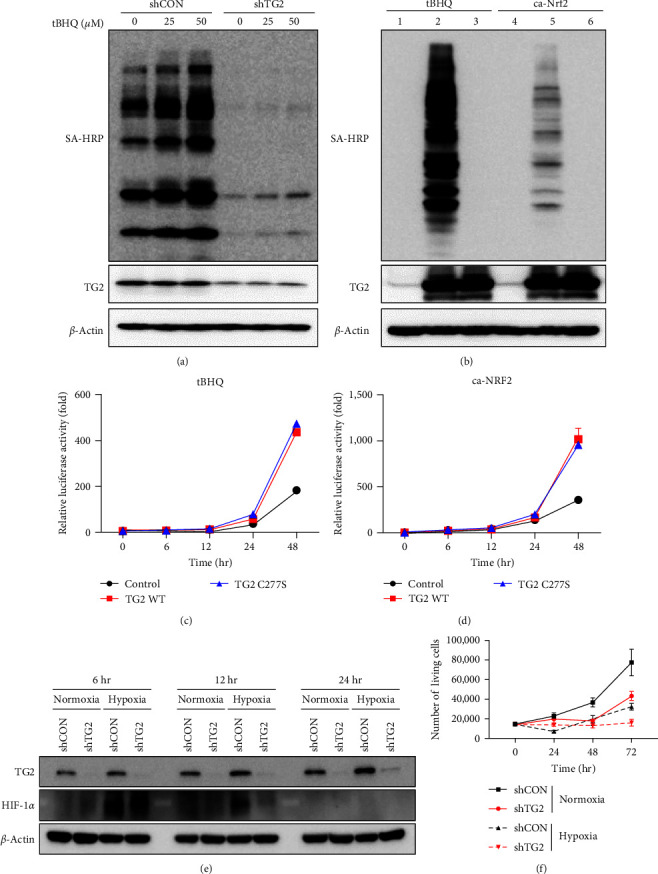
TG2 activates NRF2 in a transamidase activity-independent manner, and TG2 is required for MSCs proliferation under hypoxia conditions. (a) Wild-type and TG2-knockdowned MSCs were treated with tBHQ for 12 hr and then incubated in media containing 1 mM biotinylated pentylamine (BP) for 1 hr. BP-incorporated protein in the lysates was detected by immunoblotting with streptavidin-horseradish peroxidase (SA-HRP) to estimate intracellular transamidation activity. (b–d) HEK293 cells were transfected with expression vectors encoding wild-type TG2 (lane 2, 5, 100 ng), TG2^C277S^, a transamidase active-site mutant (lane 3, 6, 100 ng), NRF2^*Δ*1−89^, a constitutively active NRF2 mutant (caNRF2; lane 4–6, 100 ng), NRF2 reporter (8× ARE fuzed with firefly luciferase, 150 ng), and pRL-TK (50 ng). The empty pcDNA3.1 vector was used as the control (lane 1, 4, 100 ng). After treatment with tBHQ (25 *μ*M) for 48 hr or transfection of caNRF2, intracellular transamidation activity was measured by BP-incorporation assay (b) or NRF2 reporter activity was monitored for 48 hr (c, d). Reporter activity was normalized with cotransfected Renilla luciferase activity. (e) Increase of TG2 protein levels under hypoxia. MSCs were cultured under hypoxic conditions, where the amount of oxygen was reduced to less than 1% or under normoxic conditions. The protein levels of TG2 and HIF-1*α* were analyzed by western blot with *β*-actin used as the loading control. (f) Cell proliferation of MSCs under hypoxia. MSCs were cultured under hypoxic conditions, where the amount of oxygen was reduced to less than 1% or under normoxic conditions. The trypan blue viability assay was performed on MSCs. The western blots are representative results from three independent experiments. The luciferase reporter assay and cell viability assay were performed in triplicate, with technical replicates within a single experiment, and the data are presented as the mean values ± SEM.

We next investigated TG2-mediated modification of oxidized KEAP1 that affects NRF2-KEAP1 interaction. In cancer cells, oxidative stress activates TG2, which modifies a number of proteins by catalyzing transamidation reactions, producing crosslinked or polyaminated proteins [41]. To test whether tBHQ activates TG2 in MSCs, we measured the intracellular transamidase activity of TG2 using the BP incorporation assay [42]. Treatment with tBHQ increased transamidase activity in a dose-dependent manner in control MSCs, but not in TG2-downregulated MSCs (Figure 6(a)). To establish the causal relationship between transamidase activity and NRF2 activation, we introduced wild-type TG2 and TG2^C277S^, a transamidase active-site mutant, into HEK293 cells together with a luciferase reporter fuzed with ARE. In HEK293 cells, both wild-type and TG2^C277S^ proteins were expressed at similar levels (Figure 6(b)). Treatment with tBHQ significantly increased intracellular transamidation activity in TG2-expressing cells but not in TG2^C277S^-expressing cells (Figure 6(b), left). In TG2-expressing cells, tBHQ caused a gradual increase of luciferase reporter activity in a time-dependent manner (Figure 6(c)). Under the same experimental conditions, unexpectedly, luciferase reporter activity in TG2^C277S^-expressing cells was also increased to a similar extent to that in TG2-expressing cells (Figure 6(c)), indicating that TG2 activates NRF2 independent of its transamidase activity. As previously shown (Figure 5), our results demonstrate that oxidized cysteine residues within KEAP1 are the target of TG2. Moreover, TG2 also mediates the isomerization of disulfide by catalyzing sequential oxidation and reduction of thiols (PDI activity) [43]. Thus, our results suggest that TG2 is likely to induce the conformational changes of KEAP1 through PDI activity, which thereby inhibits binding to NRF2.

In addition, to exclude the possibility that NRF2 is activated by TG2-mediated modification, we introduced NRF2^*Δ*1−89^, a constitutively active NRF2 mutant (caNRF), into HEK293 cells to test whether the transcriptional activity of NRF2 depends on TG2 level. Transfection with caNRF2 slightly increased transamidase activity in HEK293 cells expressing wild-type TG2 (Figure 6(b), right). Under these conditions, however, there was no difference in luciferase activity between wild-type and TG2^C277S^-expressing HEK293 cells (Figure 6(d)). Thus, NRF2 did not seem to be modified by TG2. Taken together, our results provide evidence that modification of oxidized cysteine residues within KEAP1 by TG2, possibly through its PDI activity, is required for NRF2 stabilization.

We previously reported that hypoxic stress upregulates TG2 expression in cancer cells through HIF1*α* binding to TG2 promoter [18]. Accordingly, we examined whether the expression levels of TG2 are upregulated in MSCs under hypoxic conditions. We found that hypoxia increased the protein levels of TG2 in MSCs after 6, 12, and 24 hr (Figure 6(e)). In this situation, the knock-down of TG2 significantly suppressed MSC proliferation (Figure 6(f)). Taken together, these data suggest that hypoxia induces the expression of TG2, which is required for MSC's proliferation under hypoxic conditions.

### 3.6. Transcriptomic Analyses Support the Role of TG2 in the Antioxidant Response and Osteoblastic Differentiation of MSCs

**Figure 7:**
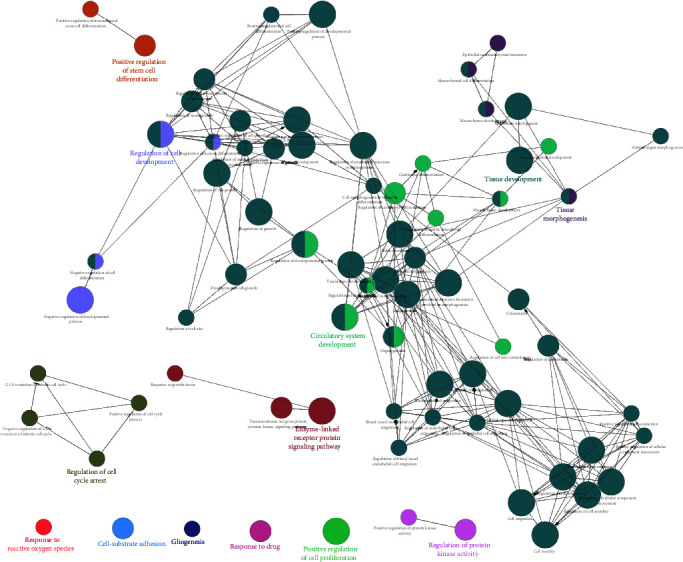
Transcriptomic analyses support the role of TG2 in antioxidant response and osteoblastic differentiation of MSCs. Network representation of enriched gene ontology (GO) biological processes (analyzed by ClueGO, *P* < 0.05) for 406 genes, which were positively correlated with TG2 expression in human bone-marrow-derived MSCs (Pearson correlation, *r* > 0.3). The transcriptome of MSCs from 61 different donors in the GEO database (GSE39540) was analyzed. The node size represents the term enrichment significance, and the nodes are linked based on their kappa score (≥0.4), where the label of the terms with the most genes per group are shown. Functionally related groups partially overlap.

To confirm the role of TG2 in MSCs, we analyzed the publicly available transcriptome of bone-marrow-derived MSC from 61 different donors in the GEO database (GSE39540). We identified 406 genes, expressions of which were positively correlated with TG2 expression (Pearson correlation, *r* > 0.3) (Table S2). When we performed GO enrichment analysis with ClueGo [29] using these 406 genes, the GO terms associated with oxidative stress (“Response to reactive oxygen species”), MSC proliferation (“Positive regulation of cell proliferation,” and “Regulation of cell cycle arrest”), and MSC differentiation (“Positive regulation of stem cell differentiation,” “Regulation of cell development,” and “Tissue morphogenesis”) were enriched (Figure 7 and Table S3). These data strengthen the promoting role of TG2 in the antioxidant response and osteoblastic differentiation of MSCs.

## 4. Discussion

The upregulated expression of TG2 in MSCs, but not in differentiated cells, suggested that TG2 may have a role in the regulation or maintenance of MSC characteristics, such as proliferation ability, surface antigen expression, or differentiation potential. In this study, we have shown that TG2-downregulated MSCs exhibit reduced proliferation and self-renewal capacity due to increased senescent cells. Moreover, TG2 is needed to promote osteoblastic but not adipocytic differentiation. In contrast, TG2 was not associated with the abilities to express MSC surface antigens and the sensitivity to oxidative stress. At the molecular level, TG2 downregulation failed to stabilize NRF2 in response to oxidative stress, leading to diminished antioxidant response. Comparing the effect of TG2 downregulation on tBHQ and SFN-induced NRF2 activity revealed that TG2 stabilizes NRF2 by modifying oxidized cysteine residues in KEAP1, and this function is independent of transamidase activity of TG2. Thus, our results indicate that TG2 plays a role in maintaining MSC proliferation and self-renewal capacities through NRF2 activation.

One of the characteristic features of stem cells, including MSCs, is their location in a hypoxic niche. MSCs are thus dependent on glycolysis to supply energy, thereby minimizing ROS production in the MSC's maintenance [44]. Previously, we showed that hypoxic stress upregulates TG2 expression in cancer cells through HIF1*α* binding to TG2 promoter [18]. Thus, the high level of TG2 expression in MSCs is likely to be attributed to their location in the hypoxic microenvironment. When mobilized to the site of tissue damage or cultured *in vitro*, MSCs are exposed to normoxia and exhibit an increased ROS production due to enhanced mitochondrial respiration [31]. Under these conditions, upregulated TG2 may contribute to protecting MSCs from functional impairment and premature senescence by eliciting NRF2-mediated antioxidant response. Indeed, transient exposure of MSCs to hypoxia enhances their therapeutic efficacy [45]. Intriguingly, TG2 inhibits ROS-induced apoptosis through caspase 3 crosslinking in cancer cells [18], suggesting a cell-type-dependent function of TG2. Therefore, our results suggest that TG2 is required for the adaptation of MSCs to oxidative stress *in vitro*, while further validation is required to confirm its *in vivo* effects within the hypoxic niche.

TG2 plays a causal role in the oxidative stress-induced inflammatory response through NF*κ*B activation [46]. In living cells, the transamidase activity of TG2 is not detected under normal conditions but increased by calcium-induced conformational changes following oxidative stress [47]. TG2 mediates crosslinking or polyamination of I*κ*B that results in its dissociation from the complex and NF*κ*B activation, eliciting an inflammatory response [48]. In this study, by contrast, we showed that TG2 augments the antioxidant response through NRF2 activation under oxidative stress conditions, implying that TG2 functions as a negative regulator in the inflammatory response by diminishing oxidative stress. Interestingly, the role of TG2 in the antioxidant response is not dependent on transamidase activity. The findings that TG2 stabilized NRF2 protein and that oxidized KEAP1 was a substrate of TG2 both suggest that the PDI activity of TG2 is likely to be involved in NRF2 activation. These results indicate that TG2 plays opposite roles in the inflammatory response to oxidative stress through differential modification of I*κ*B and KEAP1 by transamidase and PDI activity, respectively. Thus, further studies on the regulation of two enzyme activities of TG2 are needed to clarify the role of TG2 in oxidative stress-associated diseases [49].

NRF2-KEAP1 protein–protein interaction plays a critical role in the regulation of antioxidant response. The KEAP1 homodimer binds to the NRF2 through interactions between the Kelch domain (KEAP1) and low-affinity aspartate-leucine-glycine (DLG) or high-affinity glutamate-threonine-glycine-glutamate (ETGE) motifs in the Neh2 domain (NRF2), which promotes NRF2 ubiquitination and degradation [50]. Under oxidative stress conditions, ROS causes oxidation of KEAP1 cysteine residues, disrupting the Kelch-DLG but not the Kelch-ETGE interaction. According to the hinge and latch model [51], the dissociation of Kelch-DLG (latch site) inhibits KEAP1-mediated ubiquitination, and the association of Kelch-ETGE (hinge site) results in sequestration of inactivated KEAP1, which thereby allowing de novo synthesized NRF2 to stabilize, accumulate and translocate into the nucleus [51, 52]. However, the fate of the partially dissociated NRF2-KEAP1 complex remains a mystery.

Previous reports showed that H_2_O_2_ oxidizes KEAP1 cysteine residues in a nonspecific manner, generating mostly disulfide bonds [53] and that TG2 mediates the reactivation of reduced ribonuclease A, indicating that TG2 acts as a PDI [43]. We showed in this study that TG2 augments NRF2 stability and its reporter activity in response to oxidative stress in a transamidase-independent manner. Based on these findings, we propose a new model in which TG2 may induce conformational changes of KEAP1 by PDI activity-facilitated rearrangement of disulfide bonds, which disrupt Kelch-ETGE interaction. In contrast to the hinge and latch model, NRF2 is released by TG2-mediated KEAP1 modification in response to oxidative stress. Moreover, SFN induces covalent modification of KEAP1 cysteine residues, which may interfere with the PDI activity of TG2. Thus, this model explains why TG2 is required for tBHQ-, but not SFN-mediated NRF2 activation. Taken together, these results argue that TG2 is a critical regulator in the ROS-induced NRF2-KEAP1 interaction through its fail-safe mechanism.

Our results showed that TG2 is required for the differentiation of MSCs into osteoblasts but not into adipocytes. It has been reported that MSC differentiation is affected by cellular redox status [54, 55]. Induction of osteoblastic differentiation was inhibited by ROS [56] but enhanced by NRF2 overexpression [37]. In contrast, differentiation of MSCs into adipocytes was promoted by mitochondrial ROS [57], while levels of nuclear NRF2 protein and activity were diminished during MSC induction into adipocytes [58]. These results imply that TG2 promotes the osteoblastic differentiation of MSCs under oxidative stress conditions, such as inflammation, by augmenting the antioxidant response. Consistently, the role of TG2 in osteoblastic differentiation is further supported by the phenotype of TG2 knockout mice [33] and GO enrichment analysis of MSC transcriptome in the GEO database.

MSCs have both self-renewal and differentiation capacities according to the culture conditions. Although cell proliferation and differentiation usually show an inverse relationship, we found that TG2 was required for both cell proliferation and osteogenic differentiation of MSCs. Previous studies have already shown that several factors, including high glucose and zinc, promote both the proliferation and differentiation of MSCs [1, 59]. TG2 mediates post-translational modifications of diverse proteins by catalyzing transamidation or polyamination of proteins, and transamidase activity of TG2 is stimulated under a calcium-released environment [60]. Previous reports showed that extracellular calcium ions are important factors for the proliferation and migration of MSCs [61]. In addition, calcium channels and calcium signaling are involved in osteogenic differentiation of MSCs [62, 63]. One plausible explanation is that TG2 selectively modifies substrates in response to specific culture conditions, thereby promoting either cell proliferation or differentiation. Further research is required to uncover the detailed molecular mechanisms by which TG2 regulates the proliferation and differentiation of MSCs.

In recent years, MSCs have been widely used in clinical purposes and for the treatment of various diseases, including spinal cord injury, liver diseases, diabetes mellitus, brain injury, graft-versus-host disease, autoimmune diseases, and respiratory diseases [64]. As the isolated MSCs from tissues are insufficient for clinical use, large-scale expansion of MSCs *in vitro* is essential to achieve clinical applications [65]. However, the culture expansion of MSCs is accompanied by cellular senescence [4]. Therefore, the prevention of MSCs senescence is crucial during large-scale *in vitro* expansion to improve the therapeutic effect of MSCs. This study provides the first evidence that TG2 contributes as a key regulator to delay the senescence of MSCs. A high level of TG2 protects MSCs from senescence through activating NRF2, which is involved in antioxidant response. These findings suggest that TG2 is a pivotal regulator for maintaining stem cell redox homeostasis and an essential target for optimizing therapeutic applications of MSCs by preventing premature senescence.

## 5. Conclusions

We have shown that a high level of TG2 contributes to protecting MSCs from premature senescence during proliferation and to promoting osteoblastic differentiation under oxidative stress conditions by enhancing antioxidant response. Thus, TG2 provides a new target for preserving MSC function during *in vitro* MSC expansion. Because hypoxic stress upregulates TG2 expression [66], the finding that MSCs cultured under hypoxic conditions exhibit an increase in proliferation and self-renewal capacity [45] underscores the importance of TG2 in optimizing MSC manufacturing processes.

## Data Availability

The data presented in this study are available on request from the corresponding author.

## Supplementary Materials

Supplementary Materials Table S1: RT-qPCR primer sequences. Table S2: genes positively correlated with TG2 expression (*r* > 0.3). Table S3: gene ontology (GO) terms enriched in genes positively correlated with TG2 expression (*r* > 0.3).
