# Supplementary material for: Transglutaminase 2 Prevents Premature Senescence and Promotes Osteoblastic Differentiation of Mesenchymal Stem Cells through NRF2 Activation
Source: Stem Cells Int. 2023 Oct 20;2023:8815888. doi: 10.1155/2023/8815888 (PMC10611545; doi:10.1155/2023/8815888)
Supplement: Supplementary Materials — Table S1: RT-qPCR primer sequences. Table S2: genes positively correlated with TG2 expression (r > 0.3). Table S3: gene ontology (GO) terms enriched in genes positively correlated with TG2 expression (r > 0.3). [file 8815888.f1.pdf]

Supplementary Table S1. RT-qPCR primer sequences

Gene name	Forward (5′ to 3′)	Reverse (3′ to 5′)
SPP1	TTCGCAGACCTGACATCCAGTACC	TCCTCGCTTTCCATGTGTGAGG
SPARC	TGGCGAGTTTGAGAAGGTGTGC	TGTGGCAAAGAAGTGGCAGGAAG
ALP	ATCCAGATCCTGCGGAAGAACC	GTGGTCAATTCTGCCTCCTTCC
RUNX2	CACCTTGACCATAACCGTCTTCAC	CATCAAGCTTCTGTCTGTGCCTTC
PPARG	AGCCTCATGAAGAGCCTTCCAAC	TCTCCGGAAGAAACCCTTGCATC
CEBPA	AGCATTGCCTAGGAACACGAAGC	TTGTCATAACTCCGGTCCCTCTGG
FABP4	GCAGATGACAGGAAAGTCAAGAGC	TCTGCACATGTACCAGGACACC
CFD	AGGGTCACCCAAGCAACAAAGTC	ACCAACCAGATGCAGGAGTGGATG
HMOX1	TCAGGCAGAGGGTGATAGAAGAGG	TGCAACTCCTCAAAGAGCTGGATG
NQO1	TGCTGCAGCGGCTTTGAAGAAG	GCAGGGTCCTTCAGTTTACCTGTG

Supplmentary Table S2. Genes posiviely correlated with TG2 expression (*r* > 0.3)

Gene	Pearson correlation	P-value
CAPRIN2	0.666726234	4.4654E-09
GATA6	0.666478788	4.54588E-09
ITGA2	0.662797743	5.91774E-09
KRT19	0.639249909	2.94344E-08
SPOCK1	0.638154774	3.16095E-08
LIF	0.622647502	8.42311E-08
FAM8A1	0.615862492	1.27203E-07
MLLT11	0.614909739	1.34679E-07
MEST	0.612430828	1.56115E-07
RHBDF1	0.602147691	2.84279E-07
SLC20A1	0.602057285	2.85755E-07
ZMAT3	0.597402637	3.72221E-07
KRT18	0.592147345	4.99185E-07
NR2F2	0.583851965	7.85088E-07
STX1A	0.579241929	1.00432E-06
LTBP3	0.577417929	1.10595E-06
TIMP1	0.565742251	2.02224E-06
BICD1	0.561463876	2.50833E-06
LRRC17	0.556940021	3.13975E-06
ITGBL1	0.556523714	3.20477E-06
SOX9	0.556440703	3.21788E-06
CD200	0.554818077	3.48451E-06
MAP3K14	0.553375051	3.73882E-06
MICAL2	0.548731414	4.67969E-06
DNMBP	0.546998203	5.08428E-06
RGS4	0.545059723	5.57532E-06
MGP	0.540231587	6.99714E-06
ST3GAL5	0.535956956	8.53097E-06
ZNF365	0.534748455	9.01824E-06
ASAP2	0.528101409	1.21944E-05
SERPINE2	0.519101045	1.81655E-05
ARHGAP29	0.513774503	2.28766E-05
PKD2	0.51374763	2.2903E-05
TIMP3	0.513469761	2.31777E-05
EBF2	0.512306914	2.43607E-05
GULP1	0.509396726	2.7571E-05
ETV5	0.506636484	3.09737E-05
HMGA1	0.505899723	3.19455E-05
DGKI	0.503855718	3.47913E-05
CLDN14	0.503714958	3.49956E-05
IDS	0.501393668	3.85299E-05
LOC728392	0.501337539	3.86193E-05
TAX1BP1	0.493185469	5.38451E-05
WWC3	0.492944487	5.43698E-05
DUSP6	0.492328086	5.57334E-05
TNFRSF10B	0.490969043	5.88521E-05
HGF	0.488247264	6.55873E-05
PTPRK	0.486380316	7.06105E-05

HSPB8	0.485872815	7.2036E-05
LAMB1	0.484585549	7.57714E-05
APBB2	0.483054857	8.04451E-05
KDELR3	0.482099612	8.34948E-05
ATP13A3	0.480040472	9.04339E-05
ANKH	0.478419041	9.62671E-05
KCNN4	0.477262425	0.000100637
WWTR1	0.476399039	0.000104018
GLIPR1	0.475790285	0.000106464
MOGS	0.475070473	0.000109424
DCBLD2	0.473597056	0.000115721
SLC14A1	0.472388889	0.00012113
TFPI	0.471973733	0.000123042
HOXB5	0.468779249	0.000138706
EVC	0.468247398	0.000141485
PTX3	0.467230639	0.00014694
CASK	0.467128738	0.000147498
ALPL	0.463569574	0.00016822
ZFC3H1	0.461932225	0.000178621
C1orf54	0.461657591	0.000180422
DEPTOR	0.461543433	0.000181176
PLK3	0.460602825	0.000187495
TNFAIP3	0.458511319	0.000202277
F2RL1	0.457855167	0.00020713
OSBPL10	0.457498631	0.000209811
LAMC1	0.456615635	0.000216589
HMGA2	0.452981099	0.000246647
SMURF2	0.452502439	0.000250878
SLC17A5	0.451730549	0.00025784
SVIL	0.45103589	0.000264256
MAP3K13	0.448814665	0.000285762
EFNA5	0.446626622	0.000308498
PRKCA	0.44560066	0.000319716
FAM171A1	0.44547652	0.000321099
ADD3	0.442751341	0.000352851
HSPB6	0.440015144	0.000387585
CFH	0.439912095	0.000388952
CD63	0.437099832	0.000427993
CCND1	0.436286979	0.000439923
CCL2	0.435981958	0.000444478
IGFBP3	0.433161798	0.000488661
MAN1B1	0.43199599	0.000508064
DNAJC4	0.429339309	0.000554918
ELL3	0.429244366	0.000556663
PDLIM3	0.428784039	0.000565193
SEMA3C	0.428389346	0.000572601
ZNF692	0.427928516	0.000581362
CNKSR2	0.427831547	0.000583221
TSPYL2	0.426455204	0.000610193
MTMR1	0.425901454	0.00062136

ROR1	0.423806473	0.000665304
F2R	0.423524278	0.000671434
PLK2	0.422745238	0.000688622
IFT20	0.421648781	0.000713488
NES	0.420617383	0.000737618
ZNF665	0.420226571	0.000746952
LINC01711	0.419053626	0.000775612
ZNF337	0.418997919	0.000776998
EGR3	0.416971655	0.000828943
PTHLH	0.416905602	0.000830688
ASB1	0.416904377	0.00083072
PLAT	0.416400183	0.00084415
IFNGR2	0.415773775	0.000861108
DKK1	0.415585023	0.000866278
JADE1	0.415222272	0.000876292
CDKN1A	0.413123039	0.000936326
CORO2B	0.412236742	0.000962768
AHI1	0.410601355	0.001013334
ROBO3	0.409751132	0.001040557
AHR	0.408355938	0.001086661
TRIM45	0.407404693	0.001119142
LARS	0.407282083	0.001123391
MBNL2	0.406049277	0.001166934
GALNT3	0.405870019	0.00117339
SHB	0.405796841	0.001176035
TPCN1	0.405534531	0.00118556
CES2	0.403472125	0.001262901
AFAP1	0.403465302	0.001263165
CHPF2	0.403141649	0.001275708
SLC46A3	0.402829987	0.001287892
SKIL	0.402537821	0.001299408
CDH6	0.401564228	0.001338458
SCN3A	0.400419832	0.001385703
UGGT2	0.400283025	0.00139145
TNFRSF10D	0.400213964	0.00139436
PXDC1	0.400041587	0.001401645
CAMK2N1	0.399896035	0.001407824
STARD13	0.399575098	0.001421533
PHLDA1	0.399410874	0.001428595
ZNF175	0.398939489	0.001449039
LIMK2	0.397906304	0.001494771
CFHR2	0.397791945	0.001499911
ITFG2	0.397453037	0.001515239
PRKCI	0.396090605	0.001578286
CCNL2	0.395948711	0.001584985
NT5DC3	0.39588479	0.001588012
ENTPD4	0.395781129	0.001592931
KRT23	0.394168518	0.001671232
TIAM1	0.393453016	0.001707065
DHX32	0.393042185	0.00172795

SYNJ2	0.392207949	0.001771064
GFPT2	0.391951673	0.0017845
GDF15	0.391425012	0.0018124
MICALL2	0.39047275	0.001863839
COL13A1	0.390104222	0.001884094
FCER2	0.389888958	0.001896017
SLC7A6	0.389443343	0.001920911
HTR1F	0.387711603	0.002020457
CDKN2A	0.38760149	0.002026939
PRSS3	0.38710325	0.002056506
DUSP2	0.386407081	0.002098463
TSPAN2	0.386369512	0.002100749
TNFRSF25	0.386171428	0.002112838
PIK3IP1	0.38581526	0.002134731
KHDC4	0.385608493	0.002147533
BPGM	0.385427658	0.002158786
ANXA10	0.385395251	0.002160808
STC1	0.383738399	0.002266475
DUSP3	0.38333006	0.002293216
TRIM66	0.383124508	0.002306783
GPR1	0.381354843	0.002426579
SERTAD2	0.381191555	0.002437908
HMBOX1	0.380911088	0.002457476
ZNF529	0.379945986	0.002525884
MZF1	0.379055479	0.002590505
C9orf16	0.378574877	0.002625989
CREB3L2	0.378305039	0.002646101
EDN1	0.377669246	0.002694032
MDM2	0.377547783	0.002703276
FRMD4A	0.377163399	0.002732716
DNAJB4	0.376746007	0.002765008
TNFRSF12A	0.37670505	0.002768195
MECOM	0.376343557	0.002796465
PAK3	0.376130692	0.002813232
ATP6V0A1	0.3753395	0.002876337
PERP	0.37491486	0.002910724
SLC22A1	0.374837117	0.002917058
SH3BGRL3	0.374120558	0.002976026
PLOD2	0.373446741	0.003032441
TSPAN13	0.373269636	0.003047425
ACVR1B	0.373264097	0.003047895
CDK5R1	0.373231245	0.003050682
CLUHP3	0.373090444	0.003062653
TRH	0.372380352	0.003123666
MIER2	0.371435621	0.003206511
MAPK13	0.368770678	0.003450813
BMP4	0.368201413	0.003505089
MMP24OS	0.367865795	0.003537441
TMEM159	0.367453292	0.003577568
LOC155060	0.366848125	0.003637165

ATP6V0C	0.366685665	0.003653314
C14orf132	0.36653737	0.00366811
COX7A1	0.366505107	0.003671335
DDB1	0.36585674	0.003736701
SGK1	0.365545548	0.003768438
CYP3A4	0.365289122	0.003794769
CDH2	0.365282102	0.003795492
G6PD	0.363829167	0.00394781
XPNPEP1	0.362760606	0.004063258
MAP3K7CL	0.362497085	0.004092184
VAMP1	0.362353237	0.00410805
RNFT2	0.360250786	0.004346236
PXN	0.360084882	0.00436554
LOXL2	0.359828827	0.004395483
PLAGL1	0.359536007	0.004429947
FAM155A	0.359251442	0.004463668
AKR1B10	0.359241046	0.004464904
OTUD3	0.359220546	0.004467342
FAM131A	0.357817831	0.004637019
PRAF2	0.35763302	0.004659792
ADAMTS1	0.3567033	0.004775863
GABBR1	0.356563365	0.004793551
TERF2IP	0.355286223	0.004957681
NPDC1	0.35515556	0.004974749
COPZ1	0.354433905	0.00506995
FNDC4	0.354274312	0.005091219
SLC16A4	0.354269246	0.005091896
SLC5A3	0.354259967	0.005093135
SCN8A	0.354214224	0.005099248
TNFRSF21	0.35407636	0.00511771
LOX	0.353997506	0.005128296
FGFR1	0.35347234	0.005199292
GLRX	0.353084752	0.005252243
CROCCP3	0.352586818	0.005320966
ADGRB2	0.352569584	0.005323358
RPS28	0.352087798	0.00539063
PTPRO	0.351376093	0.005491374
NRXN3	0.350892303	0.005560798
PLA1A	0.350652373	0.005595513
TSPAN5	0.349638815	0.005744273
ZNF155	0.34945343	0.005771855
ATP2C1	0.34944693	0.005772824
EFEMP1	0.349041381	0.005833577
NISCH	0.348613147	0.005898337
ANK3	0.348391662	0.005932078
PSAP	0.348122773	0.005973268
TMEM158	0.347868293	0.006012482
HOXD1	0.347654155	0.006045655
GLT8D1	0.347637777	0.006048199
GADD45A	0.347414088	0.006083035

ERVH-4	0.347391232	0.006086604
MAP3K3	0.345965518	0.00631292
PXDN	0.345818385	0.006336691
GRB14	0.345809104	0.006338193
REEP2	0.345536244	0.006382492
FICD	0.345094506	0.006454785
TNFSF4	0.344428535	0.006565131
PRSS3P2	0.343844237	0.006663302
PHLDA2	0.342831329	0.006836535
FAR2	0.342786641	0.006844268
NRCAM	0.342763214	0.006848325
CCL7	0.342191225	0.006948031
PLAG1	0.34213863	0.006957262
RNASEH2B	0.341001248	0.007159539
SEC14L2	0.340891576	0.007179313
CREB3	0.339280749	0.007475293
CDIPT	0.339239823	0.00748295
SEC14L1P1	0.338480482	0.007626261
ATP6V1A	0.338449706	0.00763212
TIPARP	0.338356493	0.007649888
PAWR	0.338099599	0.007699044
TRIM62	0.337901078	0.007737219
CDRT1	0.337891717	0.007739023
RBPMS	0.33774286	0.007767762
SHMT1	0.337563104	0.007802591
SLC25A38	0.337240119	0.007865514
BHMT2	0.337181072	0.007877065
LDOC1	0.337125524	0.007887945
MEG3	0.336809681	0.007950058
HEPH	0.336470538	0.008017228
VASH1	0.336290179	0.008053151
ANKRD1	0.335733754	0.008164862
UFM1	0.334831423	0.008348892
CSAD	0.334786736	0.008358099
DGCR11	0.333889038	0.00854494
TFPI2	0.333794036	0.008564925
GNAT3	0.333170882	0.008697022
OR2C1	0.332779261	0.008780942
PLXDC2	0.332274912	0.008890057
MET	0.331588508	0.009040454
ZNF83	0.331506713	0.009058523
IER3	0.330949769	0.009182391
GLS	0.33065648	0.00924821
PRNP	0.33054297	0.009273793
PTGIS	0.329437427	0.009526201
USP20	0.3293318	0.009550627
ETV1	0.329099743	0.009604479
NUDT3	0.329045656	0.009617068
CARD10	0.328908541	0.009649048
ATP5F1E	0.328410574	0.009765968

PKIA	0.328310115	0.009789704
ZFHX2	0.328276371	0.009797688
ANOS1	0.32806658	0.009847452
SEMA4F	0.327822001	0.009905746
VWA7	0.327445137	0.009996155
PLXNA3	0.327093463	0.010081166
LOC100288570	0.326913531	0.010124904
PLXNA2	0.326555426	0.010212441
AGAP2	0.325490532	0.010476639
POFUT2	0.325477661	0.010479868
TMEM35A	0.325099544	0.010575119
ADAMTS6	0.324879037	0.010631011
CYP1A2	0.324815279	0.01064722
RBPJ	0.324577723	0.0107078
SFTPB	0.324103023	0.01082975
GSDMB	0.324087304	0.010833809
EEF2	0.323995146	0.010857631
NCOA3	0.323205677	0.011063566
MAP2	0.323171256	0.011072622
C16orf70	0.322989492	0.011120545
GFOD2	0.322942161	0.011133054
CAPN3	0.322700162	0.0111972
SLC9A1	0.322625584	0.011217032
ZSCAN31	0.322529343	0.01124267
CPA4	0.322061192	0.011368108
PRSS12	0.322040075	0.011373795
RGS17	0.32155388	0.011505404
DDB2	0.321455662	0.01153215
DYNC1LI2	0.321220117	0.011596511
BDNF	0.320952316	0.011670063
LIPG	0.320723347	0.011733269
LINC00260	0.320580952	0.011772725
CRLF1	0.319921963	0.011956823
ALDH1A3	0.319828031	0.011983265
ABCA1	0.319535532	0.01206593
RAPGEFL1	0.319513972	0.012072042
PCDH9	0.319376203	0.012111165
ABCA8	0.319284341	0.012137312
PBXIP1	0.319136221	0.012179575
MCFD2	0.318980075	0.012224266
OSBP	0.318953438	0.012231904
THBS2	0.318439247	0.012380152
SOX5	0.318429049	0.012383108
MIA2	0.317998551	0.012508442
PRDM14	0.317724164	0.012588896
FGF2	0.316849293	0.012848407
ADAP2	0.31678814	0.012866718
RGS2	0.31652637	0.012945354
MAB21L2	0.316521959	0.012946682
MOXD1	0.31634358	0.013000509

HYMAI	0.315622991	0.013219917
LRRC40	0.314729866	0.01349628
SYT1	0.314499919	0.013568234
ZNF444	0.314465008	0.013579187
ZNF767P	0.31429054	0.013634038
FST	0.314015831	0.013720791
CLIC4	0.313686131	0.013825536
SDC4	0.31368392	0.013826241
MYH10	0.313551669	0.013868452
VEGFC	0.31342146	0.013910119
ABCC1	0.31295401	0.014060593
GDAP2	0.312181516	0.014312329
PPAT	0.311942679	0.01439094
GFI1	0.311879584	0.014411769
UBAP1	0.311611093	0.014500693
IL12A	0.311367978	0.014581618
SUSD5	0.31133814	0.014591577
TEX2	0.311271406	0.014613872
PACS1	0.311199222	0.01463802
EVI5	0.311198517	0.014638256
MMP19	0.310926654	0.014729513
TRAF1	0.31089987	0.01473853
INTS6	0.30994252	0.015063957
TAGLN3	0.309922437	0.01507085
NAT9	0.309304498	0.015284245
EBLN2	0.309165355	0.015332651
LIME1	0.309116213	0.015349779
MAST4	0.309107027	0.015352982
SNCAIP	0.308762934	0.01547339
FNDC3B	0.308719341	0.015488702
NPAS2	0.308301087	0.015636276
ZNF224	0.308027745	0.01573337
AMT	0.308012561	0.015738779
ZNF701	0.307962319	0.015756686
RGS20	0.307894899	0.015780744
NEK9	0.307544191	0.015906399
PLA2G15	0.307529939	0.015911523
ITGB1	0.30707818	0.01607469
HAGH	0.306611108	0.016244893
TRIOBP	0.306492663	0.016288299
CPS1	0.306088991	0.016436977
TNFAIP6	0.305944394	0.016490517
NIPAL3	0.305768673	0.016555781
PLTP	0.305724223	0.016572325
FLJ42627	0.305238035	0.016754206
EREG	0.305133975	0.016793356
MSX2	0.304713645	0.01695229
IMPG2	0.30456653	0.01700822
IBA57	0.303904707	0.017261786
ZNF211	0.303746499	0.017322876

ITPRID2	0.30364492	0.017362198
NSUN5P1	0.303520909	0.017410305
PDE6A	0.303437151	0.017442862
MFSD11	0.303380668	0.017464846
MTMR11	0.302640336	0.017755194
LCAT	0.301479195	0.018218875
ECE1	0.301286859	0.018296669
ZNF133	0.300315572	0.018693869

Supplementary Table S3. Gene Ontology (GO) terms enriched in genes positively correlated with TG2 expression (*r* >0.3).

GOID	GOTerm	Term PValue	Term PValue Corrected with Bonferroni step down	Group PValue	Group PValue Corrected with Bonferroni step down	GOLevels	GOGroups	% Associated Genes	No. of Genes	Associated Genes Found
GO:0000302	response to reactive oxygen species	0.00	0.03	0.00	0.00	[4]	Group00	5.96	18.00	[CDKN1A, CDKN2A, EDN1, EEF2, HGF, MAPK13, MDM2, MECOM, MET, PAWR, PKD2, PLK3, PSAP, PTPRK, PXDN, PXN, TFPI2, TNFAIP3]
GO:0008284	positive regulation of cell proliferation	0.00	0.00	0.00	0.00	[2, 3, 4, 5]	Group01	4.39	51.00	[ABCC1, ADAMTS1, AGAP2, ASAP2, BMP4, CCND1, CD200, CDKN1A, CDKN2A, CRLF1, EDN1, EGR3, ELL3, EREG, ETV5, F2R, FGF2, FGFR1, GATA6, GDF15, HGF, HMGA2, IL12A, ITGA2, ITGB1, LAMB1, LAMC1, LIF, LTBP3, MAB21L2, MAP3K3, MDM2, NES, NR2F2, PKD2, PLAG1, PLOD2, PRKCA, PRKCI, PTHLH, PTX3, RBPJ, RNASEH2B, SLC20A1, SOX9, TIAM1, TIMP1, TNFAIP3, TNFSF4 VEGFC, WWTR1]
GO:0031589	cell-substrate adhesion	0.00	0.00	0.00	0.00	[3]	Group02	5.83	25.00	[CASK, CD63, CDKN2A, COL13A1, DUSP3, EFNA5, ITGA2, ITGB1, LAMB1, LAMC1, MAP3K14, MECOM, MICALL2, NCOA3, PTPRK, PTPRO, PXDN, PXN, SDC4, SLC9A1, SPOCK1, TIAM1, TNFRSF12A, TRIOBP, VEGFC]
GO:0042063	gliogenesis	0.00	0.02	0.00	0.00	[5, 6, 7]	Group03	5.75	20.00	[CCL2, CDH2, CDK5R1, CDKN1A, EEF2, ETV5, LAMB1, LAMC1, LIF, PLAG1, PRKCI, PTHLH, PTX3, ROR1, SERPINE2, SOX9, TIAM1, TNFRSF21, TSPAN2, ZNF365]
GO:0042493	response to drug	0.00	0.00	0.00	0.00	[3]	Group04	4.08	53.00	[ABCA1, ABCA8, ABCC1, AHR, ANKRD1, CCL7, CCND1, CDK5R1, CDKN1A, CDKN2A, CPS1, CYP1A2, CYP3A4, DUSP6, EDN1, EEF2, EFNA5, F2R, F2RL1, G6PD, GATA6, GNAT3, HGF, HMGA2, ITGA2, LDOC1, LOX, MAPK13, MDM2, MECOM, MET, PAK3, PAWR, PPAT, PRAF2, PRNP, PSAP, PTHLH, PTX3, RGS17, RGS2, RGS4, SEMA3C, SLC22A1, SLC25A38, SOX9, SYT1, TFPI2, TIAM1, TIMP1, TNFAIP3, TRH, VEGFC]
GO:2000738	positive regulation of stem cell differentiation	0.00	0.00	0.00	0.00	[4, 5, 6]	Group05	27.27	6.00	[BMP4, GATA6, LTBP3, SEMA3C, SOX5, SOX9]
GO:2000741	positive regulation of mesenchymal stem cell differentiation	0.00	0.04	0.00	0.00	[5, 6, 7]	Group05	60.00	3.00	[LTBP3, SOX5, SOX9]
GO:0045859	regulation of protein kinase activity	0.00	0.00	0.00	0.00	[6, 7, 8, 9]	Group06	4.20	45.00	[AGAP2, BDNF, BMP4, CAMK2N1, CARD10, CCND1, CCNL2, CDK5R1, CDKN1A, CDKN2A, DEPTOR, DKK1, DUSP2, DUSP3, DUSP6, EDN1, EFNA5, EREG, F2R, FGF2, FGFR1, GADD45A, GDF15, HGF, MAP3K13, MAP3K14, MAP3K3, MAP3K7CL, MECOM, NR2F2, PAK3, PIK3IP1, PKD2, PKIA, PRKCA, PRNP, RGS2, RGS4, SDC4, TIAM1, TIMP1, TNFAIP3, TNFRSF10B, TSPYL2, WWTR1]
GO:0045860	positive regulation of protein kinase activity	0.00	0.02	0.00	0.00	[7, 8, 9, 10]	Group06	4.57	32.00	[AGAP2, BDNF, BMP4, CARD10, CCND1, CCNL2, CDK5R1, CDKN1A, DKK1, DUSP6, EDN1, EFNA5, EREG, F2R, FGF2, FGFR1, GADD45A, GDF15, HGF, MAP3K13, MAP3K14, MAP3K3, MAP3K7CL, MECOM, PAK3, PKD2, PRKCA, PRNP, SDC4, TIAM1, TIMP1, TNFRSF10B]
GO:0070848	response to growth factor	0.00	0.02	0.00	0.00	[4]	Group07	4.24	37.00	[ANKRD1, ANOS1, BDNF, BMP4, CASK, CCL2, CD63, CPS1, DKK1, DUSP3, DUSP6, DYNC1LI2, EDN1, EEF2, EGR3, FGF2, FGFR1, FNDC4, GALNT3, GATA6, GDF15, HGF, LTBP3, MDM2, MSX2, PKD2, PRDM14, PTPRK, PTX3, PXDN, PXN, RBPJ, SKIL, SMURF2, SOX5, SOX9, VEGFC]
GO:0007167	enzyme linked receptor protein signaling pathway	0.00	0.00	0.00	0.00	[4, 5, 6]	Group07	4.22	54.00	[ACVR1B, AHI1, ANOS1, ATP6V0A1, ATP6V0C, ATP6V1A, BDNF, BMP4, CD63, CDK5R1, DKK1, DUSP3, DUSP6, EFEMP1, EFNA5, EREG, FGF2, FGFR1, FST, GALNT3, GATA6, GDF15, GRB14, HGF, IGFBP3, LIF, LTBP3, MET, MSX2, NR2F2, PAK3, PIK3IP1, PKD2, PLAT, PRDM14, PRKCA, PTPRK, PTPRO, PXDN, PXN, RBPJ, RBPMS, RHBDF1, ROR1, SHB, SKIL, SLC20A1, SMURF2, SOX9, SYT1, TIAM1, TIPARP, VEGFC, WWTR1]
GO:0007169	transmembrane receptor protein tyrosine kinase signaling pathway	0.00	0.00	0.00	0.00	[5, 6, 7]	Group07	4.51	40.00	[AHI1, ANOS1, ATP6V0A1, ATP6V0C, ATP6V1A, BDNF, CD63, CDK5R1, DUSP3, DUSP6, EFEMP1, EFNA5, EREG, FGF2, FGFR1, GALNT3, GDF15, GRB14, HGF, IGFBP3, MET, NR2F2, PAK3, PIK3IP1, PKD2, PLAT, PRDM14, PRKCA, PXDN, PXN, RBPJ, RHBDF1, ROR1, SHB, SLC20A1, SOX9, SYT1, TIAM1, TIPARP, VEGFC]
GO:0090068	positive regulation of cell cycle process	0.00	0.03	0.00	0.00	[3, 4, 5, 6]	Group08	5.45	21.00	[ADAMTS1, CCND1, CDK5R1, CDKN1A, CDKN2A, EDN1, EREG, FGFR1, GADD45A, GATA6, HMGA2, MDM2, MSX2, PKD2, PLAGL1, PLK2, PLK3, PRKCA, RGS4, SVIL, TFPI2]
GO:0000082	G1/S transition of mitotic cell cycle	0.00	0.02	0.00	0.00	[5, 6]	Group08	5.94	19.00	[ACVR1B, ADAMTS1, CCL2, CCND1, CDKN1A, CDKN2A, CFH, GADD45A, GFI1, ITGB1, JADE1, MDM2, PKD2, PLAGL1, PLK2, PLK3, PPAT, RGS4, TFPI2]
GO:0071156	regulation of cell cycle arrest	0.00	0.01	0.00	0.00	[4, 5, 6, 7]	Group08	8.23	13.00	[CCND1, CDK5R1, CDKN1A, CDKN2A, GADD45A, GATA6, HMGA2, MDM2, PKD2, PLAGL1, PLK2, PLK3, PRKCA]
GO:2000134	negative regulation of G1/S transition of mitotic cell cycle	0.00	0.02	0.00	0.00	[6, 7, 8, 9]	Group08	8.51	12.00	[CCL2, CCND1, CDKN1A, CDKN2A, CFH, GADD45A, JADE1, MDM2, PKD2, PLAGL1, PLK2, PLK3]
GO:0051093	negative regulation of developmental process	0.00	0.00	0.00	0.00	[2, 3, 4]	Group09	4.30	51.00	[ABCA1, ADGRB2, ASAP2, BDNF, BMP4, CAPN3, CARD10, CCND1, CD200, CDK5R1, CDKN2A, DKK1, EFEMP1, EFNA5, EREG, G6PD, GDF15, GFI1, HMGA2, IER3, ITGB1, LOXL2, LRRC17, MAP2, MAP3K14, MAP3K3, MDM2, MSX2, PLK2, PLOD2, PLXNA3, PTHLH, PTPRO, PTX3, RBPJ, RGS2, RGS4, SEMA4F, SKIL, SOX9, SPOCK1, STARD13, THBS2, TIMP1, TNFRSF21, TNFSF4, TRIM62, VASH1, WWC3, WWTR1, ZNF365]
GO:0045596	negative regulation of cell differentiation	0.00	0.04	0.00	0.00	[3, 4, 5]	Group09	4.17	37.00	[ABCA1, ASAP2, BDNF, BMP4, CAPN3, CCND1, CDK5R1, CDKN2A, DKK1, EFEMP1, EFNA5, EREG, G6PD, GFI1, ITGB1, LRRC17, MAP2, MAP3K14, MDM2, MSX2, PLK2, PLOD2, PLXNA3, PTHLH, PTPRO, PTX3, RBPJ, RGS2, RGS4, SEMA4F, SKIL, SOX9, SPOCK1, TNFSF4, TRIM62, WWTR1, ZNF365]
GO:0060284	regulation of cell development	0.00	0.00	0.00	0.00	[4, 5]	Group09	4.46	50.00	[ANKRD1, BDNF, BMP4, CAPRIN2, CDH2, CDK5R1, CDKN1A, CREB3L2, DKK1, EDN1, EFNA5, ELL3, ETV5, FGFR1, G6PD, GFI1, HGF, LIF, MAP2, MAP3K13, MAP3K14, MDM2, NCOA3, NRCAM, PAK3, PLAG1, PLK2, PLOD2, PLXNA2, PLXNA3, PRKCI, PTHLH, PTPRO, PTX3, RGS2, RGS4, SEMA3C, SEMA4F, SERPINE2, SKIL, SOX9, SPOCK1, SYT1, TIAM1, TIMP1, TNFRSF12A, TNFRSF21, TRIOBP, VEGFC, ZNF365]
GO:0045664	regulation of neuron differentiation	0.00	0.05	0.00	0.00	[5, 6, 7, 8, 9]	Group09	4.27	34.00	[ANKRD1, BDNF, BMP4, CAPRIN2, CDK5R1, CREB3L2, DKK1, EFNA5, ETV5, FGFR1, GFI1, HGF, MAP2, MAP3K13, MAP3K14, MDM2, NCOA3, NRCAM, PAK3, PLK2, PLXNA2, PLXNA3, PRKCI, PTPRO, RGS2, SEMA4F, SKIL, SOX9, SPOCK1, SYT1, TIAM1, TIMP1, TNFRSF12A, ZNF365]
GO:0048729	tissue morphogenesis	0.00	0.01	0.00	0.00	[3, 4]	Group10	4.60	34.00	[AHI1, ALDH1A3, ANKRD1, BMP4, CLIC4, DKK1, EDN1, ETV5, FGF2, FGFR1, HGF, HMGA2, IFT20, ITGA2, ITGB1, LIF, MDM2, MET, MICAL2, MSX2, NR2F2, PKD2, PLOD2, POFUT2, RBPJ, ROR1, SDC4, SEMA3C, SMURF2, SOX9, STARD13, STC1, TIAM1, VEGFC]
GO:0060485	mesenchyme development	0.00	0.01	0.00	0.00	[4, 5, 6]	Group10	6.12	20.00	[BMP4, CD200, CDKN2A, EDN1, ELL3, FGFR1, HGF, HMGA2, LOXL2, MDM2, MSX2, NR2F2, PKD2, POFUT2, RBPJ, SEMA3C, SOX9, TIAM1, TRIM62, WWTR1]
GO:0048762	mesenchymal cell differentiation	0.00	0.01	0.00	0.00	[4, 5, 6, 7]	Group10	6.80	17.00	[BMP4, CDKN2A, EDN1, ELL3, FGFR1, HGF, HMGA2, LOXL2, MSX2, NR2F2, POFUT2, RBPJ, SEMA3C, SOX9, TIAM1, TRIM62, WWTR1]
GO:0001837	epithelial to mesenchymal transition	0.00	0.03	0.00	0.00	[5, 6, 7, 8]	Group10	7.74	13.00	[BMP4, CDKN2A, ELL3, HGF, HMGA2, LOXL2, MSX2, POFUT2, RBPJ, SOX9, TIAM1, TRIM62, WWTR1]
GO:0035265	organ growth	0.00	0.00	0.00	0.00	[2, 3]	Group11	8.21	17.00	[CDKN1A, DUSP6, EDN1, EVC, FGF2, FGFR1, G6PD, GATA6, MSX2, PLAG1, PSAP, RBPJ, RGS2, RGS4, SOX9, STC1, WWC3]
GO:0061061	muscle structure development	0.00	0.05	0.00	0.00	[3]	Group11	4.27	34.00	[ANKRD1, BDNF, BMP4, CAPN3, CD200, CDH2, CDKN1A, CFH, DKK1, EDN1, EEF2, EGR3, EREG, ETV1, EVC, FGF2, FGFR1, G6PD, GATA6, GDF15, IFT20, IGFBP3, ITGB1, KRT19, LIF, NR2F2, RBPJ, RGS2, RGS4, SEC14L2, SLC9A1, SOX9, SVIL, WWTR1]
GO:0048638	regulation of developmental growth	0.00	0.00	0.00	0.00	[3, 4]	Group11	6.91	27.00	[BDNF, BMP4, CAPN3, CDK5R1, CDKN1A, DUSP6, EDN1, EFNA5, FGF2, FGFR1, G6PD, GATA6, GDF15, MAP2, MAP3K13, NRCAM, PHLDA2, PLXNA2, PLXNA3, RBPJ, RGS2, RGS4, SEMA4F, SLC20A1, SYT1, TNFRSF12A, WWC3]
GO:0046620	regulation of organ growth	0.00	0.02	0.00	0.00	[3, 4, 5]	Group11	9.24	11.00	[CDKN1A, DUSP6, EDN1, FGF2, FGFR1, G6PD, GATA6, RBPJ, RGS2, RGS4, WWC3]
GO:0060537	muscle tissue development	0.00	0.01	0.00	0.00	[4]	Group11	5.22	26.00	[ANKRD1, BMP4, CAPN3, CD200, CDKN1A, DKK1, EDN1, EEF2, FGF2, FGFR1, G6PD, GATA6, IFT20, ITGB1, NR2F2, PKD2, RBPJ, RGS2, RGS4, SEC14L2, SEMA3C, SLC9A1, SOX9, SVIL, TIPARP, WWTR1]
GO:0010453	regulation of cell fate commitment	0.00	0.03	0.00	0.00	[4, 5]	Group11	17.65	6.00	[BMP4, DKK1, DUSP6, FGF2, FGFR1, RBPJ]
GO:0072359	circulatory system development	0.00	0.00	0.00	0.00	[4, 5]	Group11	4.56	59.00	[ADAMTS1, ADAMTS6, ADAP2, ADGRB2, AHI1, AHR, ANKRD1, BMP4, CARD10, CCL2, CD200, CDH2, CDKN1A, CLIC4, DKK1, DUSP6, ECE1, EDN1, EGR3, EREG, FGF2, FGFR1, G6PD, GATA6, HGF, HMGA2, HSPB6, IFT20, ITGB1, LIF, LOX, LOXL2, MAP3K3, MDM2, MICAL2, MMP19, MSX2, NR2F2, NRCAM, NRXN3, PKD2, PLK2, PRKCA, PTGIS, RBPJ, RGS2, RGS4, SEMA3C, SHB, SLC9A1, SOX9, STARD13, THBS2, TIPARP, TNFAIP3, TNFRSF12A, VASH1, VEGFC, WWTR1]
GO:0035051	cardiocyte differentiation	0.00	0.03	0.00	0.00	[4, 5, 6, 7]	Group11	7.14	14.00	[BMP4, CDKN1A, DKK1, EDN1, G6PD, GATA6, IFT20, ITGB1, NR2F2, RBPJ, RGS2, RGS4, SEMA3C, SLC9A1]
GO:1905207	regulation of cardiocyte differentiation	0.00	0.00	0.00	0.00	[4, 5, 6, 7, 8]	Group11	14.52	9.00	[BMP4, DKK1, EDN1, G6PD, GATA6, RBPJ, RGS2, RGS4, SEMA3C]
GO:2000725	regulation of cardiac muscle cell differentiation	0.00	0.02	0.00	0.00	[5, 6, 7, 8, 9, 10]	Group11	15.22	7.00	[BMP4, DKK1, EDN1, G6PD, RBPJ, RGS2, RGS4]
GO:0035265	organ growth	0.00	0.00	0.00	0.00	[2, 3]	Group12	8.21	17.00	[CDKN1A, DUSP6, EDN1, EVC, FGF2, FGFR1, G6PD, GATA6, MSX2, PLAG1, PSAP, RBPJ, RGS2, RGS4, SOX9, STC1, WWC3]
GO:0040008	regulation of growth	0.00	0.00	0.00	0.00	[2, 3]	Group12	5.10	45.00	[ACVR1B, APBB2, BDNF, BMP4, CAPN3, CAPRIN2, CDK5R1, CDKN1A, CDKN2A, CFH, DCBLD2, DUSP6, EDN1, EFNA5, FGF2, FGFR1, G6PD, GATA6, GDF15, HMGA2, IGFBP3, ITPRID2, JADE1, MAP2, MAP3K13, NRCAM, OSBP, PHLDA2, PLXNA2, PLXNA3, RBPJ, RGS2, RGS4, SEMA4F, SERPINE2, SERTAD2, SGK1, SLC20A1, SLC9A1, SPOCK1, SYT1, TNFRSF12A, TSPYL2, WWC3, ZMAT3
GO:0040012	regulation of locomotion	0.00	0.00	0.00	0.00	[2, 3]	Group12	4.55	54.00	[ACVR1B, ADAMTS1, BMP4, CARD10, CCL2, CCL7, CD200, CLIC4, CREB3, DDB1, DUSP3, EDN1, EREG, F2R, F2RL1, FGF2, FGFR1, HGF, IGFBP3, IL12A, ITGA2, LAMB1, MAP3K14, MAP3K3, MDM2, MECOM, MET, NISCH, NR2F2, PAK3, PHLDA2, PKD2, PLK2, PLXNA2, PLXNA3, PRDM14, PRKCA, PTPRK, PTPRO, SDC4, SEMA3C, SERPINE2, SGK1, SMURF2, SOX9, STARD13, STC1, TFPI2, TIAM1, TIMP1, TNFAIP6, TRIM62, VASH1, VEGFC]
GO:0048870	cell motility	0.00	0.00	0.00	0.00	[2, 3]	Group12	4.06	80.00	[ABCC1, ACVR1B, ADAMTS1, APBB2, BMP4, CARD10, CCL2, CCL7, CD200, CD63, CDH2, CDK5R1, CDKN1A, CLIC4, CREB3, DUSP3, EDN1, EGR3, EREG, F2R, F2RL1, FGF2, FGFR1, GRB14, HGF, IGFBP3, IL12A, ITGA2, ITGB1, LAMB1, LAMC1, LOXL2, MAP3K14, MAP3K3, MDM2, MECOM, MET, MSX2, NISCH, NR2F2, NRCAM, PAK3, PHLDA2, PKD2, PLAT, PLK2, PLTP, PLXNA2, PRDM14, PRKCA, PRKCI, PRSS3, PRSS3P2, PTPRK, PTPRO, PXDN, PXN, RHBDF1, SDC4, SEMA3C, SERPINE2, SGK1, SLC16A4, SLC7A6, SLC9A1, SMURF2, SOX9, SPOCK1, STARD13, STC1, TFPI2, TIAM1, TIMP1, TNFAIP6, TNFRSF10B, TNFRSF10D, TNFRSF12A, TPCN1, VASH1, VEGFC]
GO:0009888	tissue development	0.00	0.00	0.00	0.00	[3]	Group12	4.23	97.00	[ACVR1B, AGAP2, AHI1, ALDH1A3, ALPL, ANKH, ANKRD1, ASAP2, ATP2C1, BMP4, CAPN3, CCND1, CD200, CDH2, CDKN1A, CDKN2A, CES2, CLIC4, CPS1, CREB3L2, CRLF1, DKK1, DUSP2, DUSP6, EBF2, EDN1, EEF2, EFEMP1, ELL3, EREG, ETV5, EVC, F2RL1, FGF2, FGFR1, FST, G6PD, GATA6, HGF, HMGA2, HOXB5, IER3, IFT20, ITGA2, ITGB1, KCNN4, KRT18, KRT19, KRT23, LAMB1, LAMC1, LIF, LOXL2, LRRC17, MDM2, MEST, MET, MGP, MICAL2, MSX2, NCOA3, NR2F2, PERP, PHLDA2, PKD2, PLOD2, PLXNA2, POFUT2, PSAP, PTGIS, PTHLH, PTPRO, PTX3, RBPJ, RGS2, RGS4, ROR1, SDC4, SEC14L2, SEMA3C, SERPINE2, SKIL, SLC20A1, SLC9A1, SMURF2, SOX5, SOX9, STARD13, STC1, SVIL, TIAM1, TIMP1, TIPARP, TRIM62, TRIOBP, VEGFC, WWTR1]
GO:0040017	positive regulation of locomotion	0.00	0.03	0.00	0.00	[2, 3, 4]	Group12	4.52	31.00	[ACVR1B, ADAMTS1, BMP4, CCL7, CREB3, DDB1, EDN1, F2R, F2RL1, FGF2, FGFR1, HGF, IL12A, ITGA2, LAMB1, MAP3K14, MAP3K3, MDM2, MECOM, MET, PAK3, PKD2, PLK2, PRDM14, PRKCA, SEMA3C, SMURF2, SOX9, TIAM1, TNFAIP6, VEGFC]
GO:0045926	negative regulation of growth	0.00	0.00	0.00	0.00	[2, 3, 4]	Group12	6.46	21.00	[ACVR1B, APBB2, BMP4, CAPRIN2, CDK5R1, CDKN1A, CDKN2A, CFH, DCBLD2, G6PD, GDF15, JADE1, MAP2, PLXNA3, RGS2, RGS4, SEMA4F, SERPINE2, SERTAD2, TSPYL2, WWC3]
GO:0048646	anatomical structure formation involved in morphogenesis	0.00	0.00	0.00	0.00	[2, 3, 4]	Group12	4.32	57.00	[ADAMTS1, ADGRB2, AHI1, ALDH1A3, ANKRD1, BMP4, CAPN3, CARD10, CCL2, CD200, CDK5R1, CLIC4, DKK1, DUSP2, DUSP6, EDN1, EGR3, EREG, FGF2, FGFR1, GATA6, GDF15, HGF, HMGA2, HSPB6, ITGA2, ITGB1, KRT19, LAMB1, LOXL2, MAP3K3, MMP19, MSX2, NR2F2, NRCAM, NRXN3, PERP, PKD2, PLK2, PLOD2, PLXNA2, POFUT2, PRDM14, PRKCA, PTGIS, RBPJ, SDC4, SEMA3C, SHB, SKIL, SOX9, STARD13, THBS2, TNFAIP3, TNFRSF12A, VASH1, VEGFC]
GO:0051094	positive regulation of developmental process	0.00	0.00	0.00	0.00	[2, 3, 4]	Group12	4.26	71.00	[ACVR1B, ADGRB2, AGAP2, AHI1, ANKRD1, BDNF, BMP4, CAPN3, CAPRIN2, CCND1, CDH2, CDKN1A, CDKN2A, CREB3L2, DKK1, EDN1, EFNA5, EGR3, ELL3, ETV5, FGF2, FGFR1, FST, GATA6, GDF15, HGF, HMGA1, HMGA2, HSPB6, IGFBP3, IL12A, ITGB1, ITPRID2, LIF, LOXL2, LTBP3, MAP3K13, MAP3K3, MSX2, NCOA3, NRCAM, PAK3, PAWR, PKD2, PLAG1, PLK2, PRDM14, PRKCA, PRKCI, PTGIS, PTX3, RBPJ, RGS2, SEMA3C, SERPINE2, SKIL, SLC20A1, SMURF2, SOX5, SOX9, SYT1, THBS2, TIAM1, TIMP1, TNFAIP3, TNFRSF12A, TNFSF4, TRIOBP, VEGFC, WWTR1, ZNF365]
GO:0006935	chemotaxis	0.00	0.00	0.00	0.00	[3, 4]	Group12	4.77	36.00	[ABCC1, ANOS1, APBB2, BDNF, BMP4, CCL2, CCL7, CDK5R1, CREB3, DUSP3, EDN1, EFNA5, EGR3, ETV1, F2RL1, FGF2, FGFR1, HGF, IL12A, ITGA2, LAMC1, MECOM, MET, NRCAM, NRXN3, PKD2, PLOD2, PLXNA2, PLXNA3, PRKCA, PTPRO, ROBO3, SEMA3C, SEMA4F, TIAM1, VEGFC]
GO:0016477	cell migration	0.00	0.00	0.00	0.00	[3, 4]	Group12	4.23	76.00	[ABCC1, ACVR1B, ADAMTS1, APBB2, BMP4, CARD10, CCL2, CCL7, CD200, CD63, CDH2, CDK5R1, CDKN1A, CLIC4, CREB3, DUSP3, EDN1, EGR3, F2R, F2RL1, FGF2, FGFR1, GRB14, HGF, IGFBP3, IL12A, ITGA2, ITGB1, LAMB1, LAMC1, LOXL2, MAP3K14, MAP3K3, MDM2, MECOM, MET, MSX2, NISCH, NR2F2, NRCAM, PAK3, PHLDA2, PKD2, PLAT, PLK2, PLXNA2, PRKCA, PRKCI, PRSS3, PRSS3P2, PTPRK, PTPRO, PXDN, PXN, RHBDF1, SDC4, SEMA3C, SERPINE2, SGK1, SLC16A4, SLC7A6, SLC9A1, SMURF2, SOX9, SPOCK1, STARD13, STC1, TFPI2, TIAM1, TIMP1, TNFAIP6, TNFRSF10B, TNFRSF10D, TNFRSF12A, VASH1, VEGFC]

GO:0022603	regulation of anatomical structure morphogenesis	0.00	0.00	0.00	0.00	[3, 4]	Group12	4.43	54.00	[ADGRB2, AHI1, BDNF, BMP4, CAPRIN2, CARD10, CCL2, CCL7, CD200, CDK5R1, DKK1, DUSP6, EDN1, EFNA5, ETV5, FGF2, FGFR1, GATA6, GDF15, HGF, HMGA2, HSPB6, ITGB1, LIF, MAP2, MAP3K13, MAP3K3, MYH10, NCOA3, NRCAM, PAK3, PKD2, PLK2, PLXNA2, PLXNA3, PRKCA, PTGIS, PTPRO, RBPJ, ROR1, SEMA3C, SEMA4F, SKIL, SMURF2, SOX9, STARD13, SYT1, THBS2, TIAM1, TNFAIP3, TNFRSF12A, TRIOBP, VASH1, VEGFC]
GO:0035295	tube development	0.00	0.00	0.00	0.00	[3, 4]	Group12	4.35	54.00	[ADGRB2, AHI1, BMP4, CARD10, CCL2, CD200, CDH2, CDKN1A, CLIC4, CPS1, CRLF1, CYP1A2, EDN1, EGR3, EREG, ETV5, FGF2, FGFR1, GATA6, HGF, HMGA2, HSPB6, ITGB1, LIF, LOX, LOXL2, MAP3K3, MET, MICAL2, MMP19, MSX2, NR2F2, NRCAM, NRXN3, PKD2, PLK2, PLOD2, PLXNA2, PRKCA, PTGIS, RBPJ, SDC4, SEMA3C, SERPINE2, SHB, SOX9, STARD13, THBS2, TIPARP, TNFAIP3, TNFRSF12A, VASH1, VEGFC, WWTR1]
GO:0048638	regulation of developmental growth	0.00	0.00	0.00	0.00	[3, 4]	Group12	6.91	27.00	[BDNF, BMP4, CAPN3, CDK5R1, CDKN1A, DUSP6, EDN1, EFNA5, FGF2, FGFR1, G6PD, GATA6, GDF15, MAP2, MAP3K13, NRCAM, PHLDA2, PLXNA2, PLXNA3, RBPJ, RGS2, RGS4, SEMA4F, SLC20A1, SYT1, TNFRSF12A, WWC3]
GO:0048729	tissue morphogenesis	0.00	0.01	0.00	0.00	[3, 4]	Group12	4.60	34.00	[AHI1, ALDH1A3, ANKRD1, BMP4, CLIC4, DKK1, EDN1, ETV5, FGF2, FGFR1, HGF, HMGA2, IFT20, ITGA2, ITGB1, LIF, MDM2, MET, MICAL2, MSX2, NR2F2, PKD2, PLOD2, POFUT2, RBPJ, ROR1, SDC4, SEMA3C, SMURF2, SOX9, STARD13, STC1, TIAM1, VEGFC]
GO:0051270	regulation of cellular component movement	0.00	0.00	0.00	0.00	[3, 4]	Group12	4.47	53.00	[ACVR1B, ADAMTS1, BMP4, CARD10, CCL2, CCL7, CD200, CLIC4, CREB3, DUSP3, EDN1, EREG, F2R, F2RL1, FGF2, FGFR1, HGF, IGFBP3, IL12A, ITGA2, LAMB1, MAP2, MAP3K14, MAP3K3, MDM2, MECOM, MET, NISCH, NR2F2, PAK3, PHLDA2, PKD2, PLK2, PLXNA2, PLXNA3, PRDM14, PRKCA, PTPRK, PTPRO, SDC4, SEMA3C, SERPINE2, SGK1, SMURF2, SOX9, STARD13, STC1, TFPI2, TIAM1, TIMP1, TNFAIP6, VASH1, VEGFC]
GO:0001558	regulation of cell growth	0.00	0.00	0.00	0.00	[3, 4, 5]	Group12	5.75	31.00	[ACVR1B, APBB2, BDNF, CAPRIN2, CDK5R1, CDKN1A, CDKN2A, CFH, DCBLD2, EDN1, EFNA5, G6PD, IGFBP3, ITPRID2, JADE1, MAP2, MAP3K13, NRCAM, PLXNA2, PLXNA3, RGS2, RGS4, SEMA4F, SERPINE2, SERTAD2, SGK1, SLC9A1, SPOCK1, SYT1, TNFRSF12A, TSPYL2]
GO:0035239	tube morphogenesis	0.00	0.00	0.00	0.00	[3, 4, 5]	Group12	4.42	45.00	[ADGRB2, AHI1, BMP4, CARD10, CCL2, CD200, CDH2, CLIC4, EDN1, EGR3, EREG, ETV5, FGF2, FGFR1, GATA6, HGF, HMGA2, HSPB6, ITGB1, LOX, LOXL2, MAP3K3, MET, MICAL2, MMP19, MSX2, NR2F2, NRCAM, NRXN3, PKD2, PLK2, PLOD2, PRKCA, PTGIS, RBPJ, SDC4, SHB, SOX9, STARD13, THBS2, TIPARP, TNFAIP3, TNFRSF12A, VASH1, VEGFC]
GO:0045596	negative regulation of cell differentiation	0.00	0.04	0.00	0.00	[3, 4, 5]	Group12	4.17	37.00	[ABCA1, ASAP2, BDNF, BMP4, CAPN3, CCND1, CDK5R1, CDKN2A, DKK1, EFEMP1, EFNA5, EREG, G6PD, GFI1, ITGB1, LRRC17, MAP2, MAP3K14, MDM2, MSX2, PLK2, PLOD2, PLXNA3, PTHLH, PTPRO, PTX3, RBPJ, RGS2, RGS4, SEMA4F, SKIL, SOX9, SPOCK1, TNFSF4, TRIM62, WWTR1, ZNF365]
GO:0045597	positive regulation of cell differentiation	0.00	0.01	0.00	0.00	[3, 4, 5]	Group12	4.00	48.00	[ACVR1B, AHI1, ANKRD1, BDNF, BMP4, CAPRIN2, CDH2, CREB3L2, DKK1, EDN1, EFNA5, EGR3, ELL3, ETV5, FGF2, FGFR1, GATA6, GDF15, HGF, IGFBP3, IL12A, LIF, LOXL2, LTBP3, MAP3K13, MSX2, NCOA3, NRCAM, PAK3, PLAG1, PRKCA, PRKCI, RBPJ, RGS2, SEMA3C, SERPINE2, SKIL, SOX5, SOX9, SYT1, TIAM1, TIMP1, TNFRSF12A, TNFSF4, TRIOBP, VEGFC, WWTR1, ZNF365]
GO:0046620	regulation of organ growth	0.00	0.02	0.00	0.00	[3, 4, 5]	Group12	9.24	11.00	[CDKN1A, DUSP6, EDN1, FGF2, FGFR1, G6PD, GATA6, RBPJ, RGS2, RGS4, WWC3]
GO:0048588	developmental cell growth	0.00	0.00	0.00	0.00	[3, 4, 5]	Group12	7.14	20.00	[BDNF, CDK5R1, EDN1, EFNA5, G6PD, ITGB1, LAMC1, MAP2, MAP3K13, NRCAM, PLOD2, PLXNA2, PLXNA3, RGS2, RGS4, SEMA4F, SOX9, SYT1, TIAM1, TNFRSF12A]
GO:0050920	regulation of chemotaxis	0.00	0.00	0.00	0.00	[3, 4, 5]	Group12	7.06	18.00	[CCL2, CCL7, CREB3, DUSP3, EDN1, F2RL1, FGF2, FGFR1, IL12A, ITGA2, MECOM, MET, PKD2, PLXNA2, PLXNA3, PTPRO, TIAM1, VEGFC]
GO:0051272	positive regulation of cellular component movement	0.00	0.02	0.00	0.00	[3, 4, 5]	Group12	4.67	31.00	[ACVR1B, ADAMTS1, BMP4, CCL7, CREB3, EDN1, F2R, F2RL1, FGF2, FGFR1, HGF, IL12A, ITGA2, LAMB1, MAP2, MAP3K14, MAP3K3, MDM2, MECOM, MET, PAK3, PKD2, PLK2, PRDM14, PRKCA, SEMA3C, SMURF2, SOX9, TIAM1, TNFAIP6, VEGFC]
GO:0060429	epithelium development	0.00	0.00	0.00	0.00	[4]	Group12	4.09	60.00	[ACVR1B, AGAP2, AHI1, ALDH1A3, ASAP2, BMP4, CCND1, CD200, CDH2, CDKN1A, CES2, CLIC4, CPS1, CRLF1, DKK1, EDN1, EREG, ETV5, F2RL1, FGF2, FGFR1, FST, GATA6, HGF, HOXB5, IFT20, ITGA2, KRT18, KRT19, KRT23, LAMC1, LIF, LOXL2, MET, MICAL2, MSX2, NCOA3, NR2F2, PERP, PKD2, PLOD2, PLXNA2, PSAP, PTPRO, PTX3, RBPJ, ROR1, SDC4, SEC14L2, SEMA3C, SERPINE2, SKIL, SMURF2, SOX9, STARD13, STC1, TIAM1, TRIOBP, VEGFC, WWTR1]
GO:0060537	muscle tissue development	0.00	0.01	0.00	0.00	[4]	Group12	5.22	26.00	[ANKRD1, BMP4, CAPN3, CD200, CDKN1A, DKK1, EDN1, EEF2, FGF2, FGFR1, G6PD, GATA6, IFT20, ITGB1, NR2F2, PKD2, RBPJ, RGS2, RGS4, SEC14L2, SEMA3C, SLC9A1, SOX9, SVIL, TIPARP, WWTR1]
GO:2000145	regulation of cell motility	0.00	0.00	0.00	0.00	[3, 4, 5]	Group12	4.64	51.00	[ACVR1B, ADAMTS1, BMP4, CARD10, CCL2, CCL7, CD200, CLIC4, CREB3, DUSP3, EDN1, EREG, F2R, F2RL1, FGF2, FGFR1, HGF, IGFBP3, IL12A, ITGA2, LAMB1, MAP3K14, MAP3K3, MDM2, MECOM, MET, NISCH, NR2F2, PAK3, PHLDA2, PKD2, PLK2, PLXNA2, PRDM14, PRKCA, PTPRK, PTPRO, SDC4, SEMA3C, SERPINE2, SGK1, SMURF2, SOX9, STARD13, STC1, TFPI2, TIAM1, TIMP1, TNFAIP6, VASH1, VEGFC]
GO:0001667	ameboidal-type cell migration	0.00	0.00	0.00	0.00	[4, 5]	Group12	6.54	32.00	[ACVR1B, BMP4, CARD10, CD200, EDN1, EGR3, FGF2, FGFR1, ITGA2, ITGB1, LOXL2, MAP3K14, MAP3K3, MET, NR2F2, PAK3, PKD2, PLK2, PRKCA, PRSS3, PRSS3P2, PXDN, PXN, SDC4, SEMA3C, SMURF2, SOX9, STARD13, STC1, TIMP1, VASH1, VEGFC]
GO:0008361	regulation of cell size	0.00	0.04	0.00	0.00	[4, 5]	Group12	7.07	14.00	[BDNF, CDK5R1, DEPTOR, EDN1, EFNA5, KCNN4, LARS, MAP2, MAP3K13, NRCAM, PLXNA2, PLXNA3, SEMA4F, TNFRSF12A]
GO:0009887	animal organ morphogenesis	0.00	0.01	0.00	0.00	[3, 4, 5, 6]	Group12	4.04	47.00	[ADAMTS1, AHI1, ALDH1A3, ALPL, ANKRD1, BMP4, CCL2, CDH2, CFH, COL13A1, DKK1, EDN1, EFEMP1, EREG, ETV5, FGF2, FGFR1, GATA6, HGF, HOXB5, ITGA2, LAMB1, LIF, MDM2, MGP, MICAL2, MSX2, NR2F2, PERP, PHLDA2, PKD2, PLAG1, PRKCI, PTX3, RBPJ, ROR1, SEMA3C, SFTPB, SMURF2, SOX9, STC1, TIAM1, TIPARP, TNFAIP3, TRIOBP, VEGFC, WWTR1]
GO:0030308	negative regulation of cell growth	0.00	0.00	0.00	0.00	[3, 4, 5, 6]	Group12	7.86	18.00	[ACVR1B, APBB2, CAPRIN2, CDK5R1, CDKN1A, CDKN2A, CFH, DCBLD2, G6PD, JADE1, MAP2, PLXNA3, RGS2, RGS4, SEMA4F, SERPINE2, SERTAD2, TSPYL2]
GO:0060284	regulation of cell development	0.00	0.00	0.00	0.00	[4, 5]	Group12	4.46	50.00	[ANKRD1, BDNF, BMP4, CAPRIN2, CDH2, CDK5R1, CDKN1A, CREB3L2, DKK1, EDN1, EFNA5, ELL3, ETV5, FGFR1, G6PD, GFI1, HGF, LIF, MAP2, MAP3K13, MAP3K14, MDM2, NCOA3, NRCAM, PAK3, PLAG1, PLK2, PLOD2, PLXNA2, PLXNA3, PRKCI, PTHLH, PTPRO, PTX3, RGS2, RGS4, SEMA3C, SEMA4F, SERPINE2, SKIL, SOX9, SPOCK1, SYT1, TIAM1, TIMP1, TNFRSF12A, TNFRSF21, TRIOBP, VEGFC, ZNF365]
GO:0072359	circulatory system development	0.00	0.00	0.00	0.00	[4, 5]	Group12	4.56	59.00	[ADAMTS1, ADAMTS6, ADAP2, ADGRB2, AHI1, AHR, ANKRD1, BMP4, CARD10, CCL2, CD200, CDH2, CDKN1A, CLIC4, DKK1, DUSP6, ECE1, EDN1, EGR3, EREG, FGF2, FGFR1, G6PD, GATA6, HGF, HMGA2, HSPB6, IFT20, ITGB1, LIF, LOX, LOXL2, MAP3K3, MDM2, MICAL2, MMP19, MSX2, NR2F2, NRCAM, NRXN3, PKD2, PLK2, PRKCA, PTGIS, RBPJ, RGS2, RGS4, SEMA3C, SHB, SLC9A1, SOX9, STARD13, THBS2, TIPARP, TNFAIP3, TNFRSF12A, VASH1, VEGFC, WWTR1]
GO:0000904	cell morphogenesis involved in differentiation	0.00	0.02	0.00	0.00	[4, 5, 6]	Group12	4.30	37.00	[ANK3, ANOS1, APBB2, BDNF, CAPRIN2, CDK5R1, CLIC4, DKK1, EFNA5, ETV1, IER3, ITGB1, LAMB1, LAMC1, MAP2, MAP3K13, MET, MICALL2, NCOA3, NRCAM, NRXN3, PAK3, PLOD2, PLXNA2, PLXNA3, PRKCA, PTPRO, ROBO3, SEC14L2, SEMA3C, SEMA4F, SKIL, STC1, TIAM1, TNFRSF12A, TRIOBP, ZNF365]
GO:0010631	epithelial cell migration	0.00	0.00	0.00	0.00	[4, 5, 6]	Group12	6.95	26.00	[BMP4, CARD10, CD200, EDN1, EGR3, FGF2, FGFR1, ITGA2, ITGB1, LOXL2, MAP3K14, MAP3K3, MET, NR2F2, PKD2, PLK2, PRKCA, PRSS3, PRSS3P2, PXDN, PXN, SOX9, STARD13, STC1, VASH1, VEGFC]
GO:0010632	regulation of epithelial cell migration	0.00	0.01	0.00	0.00	[3, 4, 5, 6, 7]	Group12	6.64	19.00	[BMP4, CARD10, CD200, EDN1, FGF2, FGFR1, ITGA2, MAP3K14, MAP3K3, MET, NR2F2, PKD2, PLK2, PRKCA, SOX9, STARD13, STC1, VASH1, VEGFC]
GO:0030334	regulation of cell migration	0.00	0.00	0.00	0.00	[4, 5, 6]	Group12	4.73	49.00	[ACVR1B, ADAMTS1, BMP4, CARD10, CCL2, CCL7, CD200, CLIC4, CREB3, DUSP3, EDN1, F2R, F2RL1, FGF2, FGFR1, HGF, IGFBP3, IL12A, ITGA2, LAMB1, MAP3K14, MAP3K3, MDM2, MECOM, MET, NISCH, NR2F2, PAK3, PHLDA2, PKD2, PLK2, PLXNA2, PRKCA, PTPRK, PTPRO, SDC4, SEMA3C, SERPINE2, SGK1, SMURF2, SOX9, STARD13, STC1, TFPI2, TIAM1, TIMP1, TNFAIP6, VASH1, VEGFC]
GO:0051960	regulation of nervous system development	0.00	0.00	0.00	0.00	[4, 5, 6]	Group12	4.32	47.00	[ADGRB2, ANKRD1, BDNF, BMP4, CAPRIN2, CDH2, CDK5R1, CDKN1A, CREB3L2, DKK1, EFNA5, ELL3, ETV5, FGFR1, GFI1, HGF, ITPRID2, LIF, MAP2, MAP3K13, MAP3K14, MDM2, NCOA3, NRCAM, PAK3, PLAG1, PLK2, PLOD2, PLXNA2, PLXNA3, PRKCI, PTPRO, PTX3, RGS2, SEMA4F, SERPINE2, SKIL, SOX9, SPOCK1, SYT1, THBS2, TIAM1, TIMP1, TNFRSF12A, TNFRSF21, VEGFC, ZNF365]
GO:0060485	mesenchyme development	0.00	0.01	0.00	0.00	[4, 5, 6]	Group12	6.12	20.00	[BMP4, CD200, CDKN2A, EDN1, ELL3, FGFR1, HGF, HMGA2, LOXL2, MDM2, MSX2, NR2F2, PKD2, POFUT2, RBPJ, SEMA3C, SOX9, TIAM1, TRIM62, WWTR1]
GO:0001944	vasculature development	0.00	0.00	0.00	0.00	[4, 5, 6, 7]	Group12	4.92	42.00	[ADAMTS6, ADGRB2, AHR, BMP4, CARD10, CCL2, CD200, CDH2, CLIC4, EDN1, EGR3, EREG, FGF2, FGFR1, GATA6, HGF, HMGA2, HSPB6, ITGB1, LIF, LOX, LOXL2, MAP3K3, MDM2, MMP19, NR2F2, NRCAM, NRXN3, PKD2, PLK2, PRKCA, PTGIS, RBPJ, SEMA3C, SHB, STARD13, THBS2, TIPARP, TNFAIP3, TNFRSF12A, VASH1, VEGFC]
GO:0048762	mesenchymal cell differentiation	0.00	0.01	0.00	0.00	[4, 5, 6, 7]	Group12	6.80	17.00	[BMP4, CDKN2A, EDN1, ELL3, FGFR1, HGF, HMGA2, LOXL2, MSX2, NR2F2, POFUT2, RBPJ, SEMA3C, SOX9, TIAM1, TRIM62, WWTR1]
GO:0010594	regulation of endothelial cell migration	0.00	0.01	0.00	0.00	[4, 5, 6, 7, 8]	Group12	7.58	16.00	[BMP4, CARD10, CD200, EDN1, FGF2, FGFR1, MAP3K3, MET, NR2F2, PKD2, PLK2, PRKCA, STARD13, STC1, VASH1, VEGFC]
GO:0043542	endothelial cell migration	0.00	0.00	0.00	0.00	[5, 6, 7]	Group12	8.46	23.00	[BMP4, CARD10, CD200, EDN1, EGR3, FGF2, FGFR1, ITGB1, LOXL2, MAP3K3, MET, NR2F2, PKD2, PLK2, PRKCA, PRSS3, PRSS3P2, PXDN, PXN, STARD13, STC1, VASH1, VEGFC]
GO:0048666	neuron development	0.00	0.00	0.00	0.00	[4, 5, 7, 8, 9]	Group12	4.21	56.00	[AHI1, ANK3, ANKRD1, ANOS1, APBB2, BDNF, CAPRIN2, CDK5R1, CREB3L2, EFNA5, ETV1, FGFR1, GFI1, HGF, IER3, IFT20, ITGB1, LAMB1, LAMC1, LIF, MAP2, MAP3K13, MAP3K14, MDM2, MICALL2, NCOA3, NPAS2, NRCAM, NRXN3, PAK3, PLK2, PLOD2, PLXNA2, PLXNA3, PRKCA, PRKCI, PSAP, PTHLH, PTPRK, PTPRO, PTX3, RGS2, ROBO3, SDC4, SEMA3C, SEMA4F, SGK1, SKIL, SPOCK1, SYT1, TIAM1, TIMP1, TNFRSF12A, TRIOBP, TSPAN2, ZNF365]
GO:0001525	angiogenesis	0.00	0.00	0.00	0.00	[3, 4, 5, 6, 7, 8, 9, 10]	Group12	5.42	34.00	[ADGRB2, BMP4, CARD10, CCL2, CD200, CLIC4, EDN1, EGR3, EREG, FGF2, FGFR1, GATA6, HGF, HMGA2, HSPB6, ITGB1, LOXL2, MAP3K3, MMP19, NR2F2, NRCAM, NRXN3, PKD2, PLK2, PRKCA, PTGIS, RBPJ, SHB, STARD13, THBS2, TNFAIP3, TNFRSF12A, VASH1, VEGFC]
GO:0048514	blood vessel morphogenesis	0.00	0.00	0.00	0.00	[4, 5, 6, 7, 8, 9]	Group12	5.08	37.00	[ADGRB2, BMP4, CARD10, CCL2, CD200, CDH2, CLIC4, EDN1, EGR3, EREG, FGF2, FGFR1, GATA6, HGF, HMGA2, HSPB6, ITGB1, LOX, LOXL2, MAP3K3, MMP19, NR2F2, NRCAM, NRXN3, PKD2, PLK2, PRKCA, PTGIS, RBPJ, SHB, STARD13, THBS2, TIPARP, TNFAIP3, TNFRSF12A, VASH1, VEGFC]
GO:0050767	regulation of neurogenesis	0.00	0.00	0.00	0.00	[5, 6, 7, 8]	Group12	4.52	44.00	[ANKRD1, BDNF, BMP4, CAPRIN2, CDH2, CDK5R1, CDKN1A, CREB3L2, DKK1, EFNA5, ELL3, ETV5, FGFR1, GFI1, HGF, LIF, MAP2, MAP3K13, MAP3K14, MDM2, NCOA3, NRCAM, PAK3, PLAG1, PLK2, PLOD2, PLXNA2, PLXNA3, PRKCI, PTPRO, PTX3, RGS2, SEMA4F, SERPINE2, SKIL, SOX9, SPOCK1, SYT1, TIAM1, TIMP1, TNFRSF12A, TNFRSF21, VEGFC, ZNF365]
GO:0043534	blood vessel endothelial cell migration	0.00	0.01	0.00	0.00	[6, 7, 8]	Group12	8.67	13.00	[CARD10, CD200, EGR3, FGF2, FGFR1, ITGB1, MAP3K3, PKD2, PLK2, PRKCA, STARD13, VASH1, VEGFC]
GO:0043535	regulation of blood vessel endothelial cell migration	0.00	0.03	0.00	0.00	[5, 6, 7, 8, 9]	Group12	8.94	11.00	[CARD10, CD200, FGF2, FGFR1, MAP3K3, PKD2, PLK2, PRKCA, STARD13, VASH1, VEGFC]
GO:0045664	regulation of neuron differentiation	0.00	0.05	0.00	0.00	[5, 6, 7, 8, 9]	Group12	4.27	34.00	[ANKRD1, BDNF, BMP4, CAPRIN2, CDK5R1, CREB3L2, DKK1, EFNA5, ETV5, FGFR1, GFI1, HGF, MAP2, MAP3K13, MAP3K14, MDM2, NCOA3, NRCAM, PAK3, PLK2, PLXNA2, PLXNA3, PRKCI, PTPRO, RGS2, SEMA4F, SKIL, SOX9, SPOCK1, SYT1, TIAM1, TIMP1, TNFRSF12A, ZNF365]
GO:0031175	neuron projection development	0.00	0.00	0.00	0.00	[5, 6, 8, 9, 10]	Group12	4.42	52.00	[ANK3, ANKRD1, ANOS1, APBB2, BDNF, CAPRIN2, CDK5R1, CREB3L2, EFNA5, ETV1, FGFR1, GFI1, HGF, IER3, IFT20, ITGB1, LAMB1, LAMC1, MAP2, MAP3K13, MAP3K14, MDM2, MICALL2, NCOA3, NPAS2, NRCAM, NRXN3, PAK3, PLK2, PLOD2, PLXNA2, PLXNA3, PRKCA, PRKCI, PTHLH, PTPRK, PTPRO, RGS2, ROBO3, SDC4, SEMA3C, SEMA4F, SGK1, SKIL, SPOCK1, SYT1, TIAM1, TIMP1, TNFRSF12A, TRIOBP, TSPAN2, ZNF365]
GO:0010975	regulation of neuron projection development	0.00	0.01	0.00	0.00	[6, 7, 8, 9, 10, 11]	Group12	4.85	29.00	[ANKRD1, BDNF, CAPRIN2, CDK5R1, CREB3L2, EFNA5, FGFR1, GFI1, HGF, MAP2, MAP3K13, MAP3K14, MDM2, NRCAM, PAK3, PLK2, PLXNA2, PLXNA3, PRKCI, PTPRO, RGS2, SEMA4F, SKIL, SPOCK1, SYT1, TIAM1, TIMP1, TNFRSF12A, ZNF365]
